# The influence of flocculation upon global gene transcription in a yeast CYC8 mutant

**DOI:** 10.1099/mgen.0.001216

**Published:** 2024-03-26

**Authors:** Brenda Lee, Karsten Hokamp, Mohamed M. Alhussain, Atif A. Bamagoos, Alastair B. Fleming

**Affiliations:** 1 Department of Microbiology, School of Genetics and Microbiology, Moyne Institute of Preventive Medicine, Trinity College Dublin, Dublin, Ireland; 2 Department of Genetics, School of Genetics and Microbiology, Smurfit Institute, Trinity College Dublin, Dublin, Ireland; 3 Department of Biological Sciences, Faculty of Science, King Abdulaziz University, Jeddah, Saudi Arabia

**Keywords:** co-repressor, *FLO1*, flocculation, *Saccharomyces cerevisiae*, transcription, Tup1-Cyc8

## Abstract

The transcriptome from a *Saccharomyces cerevisiae tup1* deletion mutant was one of the first comprehensive yeast transcriptomes published. Subsequent transcriptomes from *tup1* and *cyc8* mutants firmly established the Tup1-Cyc8 complex as predominantly acting as a repressor of gene transcription. However, transcriptomes from *tup1/cyc8* gene deletion or conditional mutants would all have been influenced by the striking flocculation phenotypes that these mutants display. In this study, we have separated the impact of flocculation from the transcriptome in a *cyc8* conditional mutant to reveal those genes (i) subject solely to Cyc8p-dependent regulation, (ii) regulated by flocculation only and (iii) regulated by Cyc8p and further influenced by flocculation. We reveal a more accurate list of Cyc8p-regulated genes that includes newly identified Cyc8p-regulated genes that were masked by the flocculation phenotype and excludes genes which were indirectly influenced by flocculation and not regulated by Cyc8p. Furthermore, we show evidence that flocculation exerts a complex and potentially dynamic influence upon global gene transcription. These data should be of interest to future studies into the mechanism of action of the Tup1-Cyc8 complex and to studies involved in understanding the development of flocculation and its impact upon cell function.

## Data Summary

The data that support the findings of this study are publicly available from Gene Expression Omnibus (GEO) with the identifier [GSE248550], and can be visualized at https://bioinf.gen.tcd.ie/jbrowse2/?config=AA_comparisons%2Fconfig.json.

**Impact Statement**

The Tup1-Cyc8 complex in *Saccharomyces cerevisiae* is the archetypal global repressor of gene transcription. The genes regulated by Tup1-Cyc8 came from the transcriptomes of *tup1* and *cyc8* mutants which display a cell aggregation phenotype called flocculation. Flocculation is an important trait of many industrial yeast strains, including those used for brewing, and is a model system for the study of multicellularity and biofilms. We hypothesized that the transcriptomes of *tup1* and *cyc8* mutants would contain genes regulated by Tup1-Cyc8 and genes indirectly impacted by flocculation. For example, we predicted that cells within the closely packed cell aggregates would be subject to nutrient and/or oxygen limitation which would influence gene transcription. We separated the impact of flocculation from the transcriptome of a Cyc8p-deficient mutant to reveal (i) genes indirectly regulated by flocculation, (ii) genes subject solely to Cyc8p regulation and (iii) Cyc8p-regulated genes further influenced by flocculation. We present an updated list of Tup1-Cyc8-regulated genes and reveal that flocculation has a widespread and potentially dynamic influence upon global gene transcription. These data will be relevant to those studying the Tup1-Cyc8 complex, and to those interested in flocculation and its impact upon cell function, in both industry and academia.

## Introduction

The Tup1-Cyc8 co-repressor complex in the yeast *Saccharomyces cerevisiae* is the archetypal global repressor of gene transcription [1]. It is responsible for the repression of a wide variety of genes including those that respond to glucose, oxygen, DNA damage and osmotic stress [2]. Indeed, studies have shown that it is responsible for the repression of approximately 10 % of genes when yeast cells are grown in glucose [3,4].

The complex is composed of one Cyc8p (Ssn6p) subunit and four Tup1p subunits [5]. The complex does not bind to DNA itself but is recruited to target genes via DNA sequence-specific transcription factors [6,7]. The recruitment to DNA is mediated via the Cyc8p subunit whilst the Tup1p subunit was originally thought to harbour the repressive activity [8]. However, there is evidence that both subunits can occupy and influence genes independently [4].

Tup1-Cyc8 can repress genes via various mechanisms including recruiting histone deacetylases (HDACs) to target gene promoters, maintaining ordered nucleosomal arrays over promoters, directly inhibiting RNA polymerase II (RNAP II) and associated machinery, or by blocking the activation domains of transcription factors [9,20]. These mechanisms are not necessarily mutually exclusive. Evidence suggests that at some genes, repression is mediated by multiple mechanisms acting in response to the environment working either cooperatively or redundantly [21,22]. The complex has also been shown to be required for activation of some genes [4,23,25].

Most studies into Tup1-Cyc8 function have used either *TUP1* or *CYC8* deletion or conditional mutants. A striking phenotype of such mutants is their flocculation phenotype, in which cells form multi-cellular aggregates [4,26]. Flocculation is a yeast stress response whereby cells on the inside of the floc are protected from external hazardous reagents by the cells on the outside of the floc which will be exposed and which will die [27]. Thus, overall, flocculation has the potential to ensure the survival of the cell population when exposed to external stressors.

Flocculation is an asexual, calcium-dependent, reversible cell aggregation process [28]. The phenotype is determined by the expression of the *FLO* family of genes which includes the *FLO1*, *5*, *9* and *10* genes which are subject to Tup1-Cyc8-mediated repression [29]. These genes encode cell wall lectin-like proteins (flocculins) that bind to the mannose residues in the cell walls of neighbouring cells [30,31]. *FLO1* is considered to be the dominant *FLO* gene and has been well characterized [32,33]. It has been shown that Tup1-Cyc8 can bind to the *FLO1* gene promoter to bring about gene repression in an HDAC-dependent manner [9,34]. Conversely, *FLO1* activation requires Swi-Snf and histone acetylation [35,36].

The flocculins, such as Flo1p, are serine/threonine-rich proteins comprising a lectin-like N-terminus and a C-terminal glycosylphosphatidylinositol (GPI) anchor [37,40]. The central domain contains serine/threonine-rich internal repeats and is heavily O-glycosylated. The mechanism of cell aggregation requires calcium which, when bound by the flocculins, promotes a change in conformation of the Flo protein’s lectin domain to promote binding to the mannose residues in the cell walls of neighbouring cells [31,41]. Flocculation can therefore be inhibited or reversed by exposing cells to EDTA to sequester the calcium ions. Alternatively, and depending on strain background, adding excess mannose to the medium can prevent or disperse floc formation by out-competing the flocculin binding sites [42].

The flocculation phenotype is important for the brewing industry where it offers a convenient way to separate the yeast from the product at the end of the fermentation process [43]. In brewing strains, the phenotype is thought to occur in response to multiple signals including high cell density, increased ethanol concentration and glucose depletion [44,46]. Strains that flocculate are also of interest for other industrial applications of yeast due to their ability to tolerate the harsh growth conditions often employed in industrial fermentations and to aid in the downstream processing of the product [47,48]. The phenotype has also been applied to bioremediation where the *FLO* proteins, and other cell wall components, aid in the chelation and removal of heavy metal ions from industrial effluent [49,51]. Yeast flocculation is also a useful model system for the study of microbial multicellular development and biofilms [30,52,54].

In most laboratory strains of *S. cerevisiae*, flocculation is considered an undesirable trait and the phenotype has been selected against. Therefore, commonly used laboratory strains such as S288c contain a nonsense mutation in the *FLO8* gene which encodes the activator of *FLO* gene transcription [55,56]. Thus, flocculation only occurs in mutant laboratory strains, such as when *TUP1* and/or *CYC8* are deleted, and is therefore a key phenotype of cells used in most studies of Tup1-Cyc8 complex function, including those used to uncover Tup1-Cyc8-regulated genes.

In this study, we wanted to test the hypothesis that flocculation would have a significant influence upon global gene transcription due to the potential nutrient- and oxygen-depleted environment of the cells within the flocs. We have separated the impact of flocculation from the transcriptome of a Cyc8p conditional mutant and revealed (i) the genes subject solely to Cyc8p regulation, (ii) the genes specifically influenced by flocculation and (iii) Cyc8p-dependent genes which are further positively or negatively influenced by flocculation. Together, we offer a revised profile of Cyc8p-regulated genes and reveal that flocculation exerts a widespread and complex influence upon global gene transcription.

## Methods

### Yeast strains and growth conditions

The Cyc8p anchor-away strain (Cyc8-AA; Mata *tor1-1 fpr1*::loxP-*LEU2*-loxP *RPL13A2*×FKBP12::loxP *CYC8*-FRB::*HIS3*) used throughout this study was constructed in the HHY221 background strain as previously reported [35,57]. Cells were grown at 30 °C in Yeast Extract-Peptone-Dextrose (YPD) medium unless stated otherwise.

### Flocculation assay

This assay was performed as previously described [4,35]. Cells with a flocculant phenotype were dispersed in the presence of EDTA (20 mM). Cells grown in the presence of mannose (250 mM) for up to 4 h do not flocculate.

### Anchor-away experiments

**Fig. 1.**
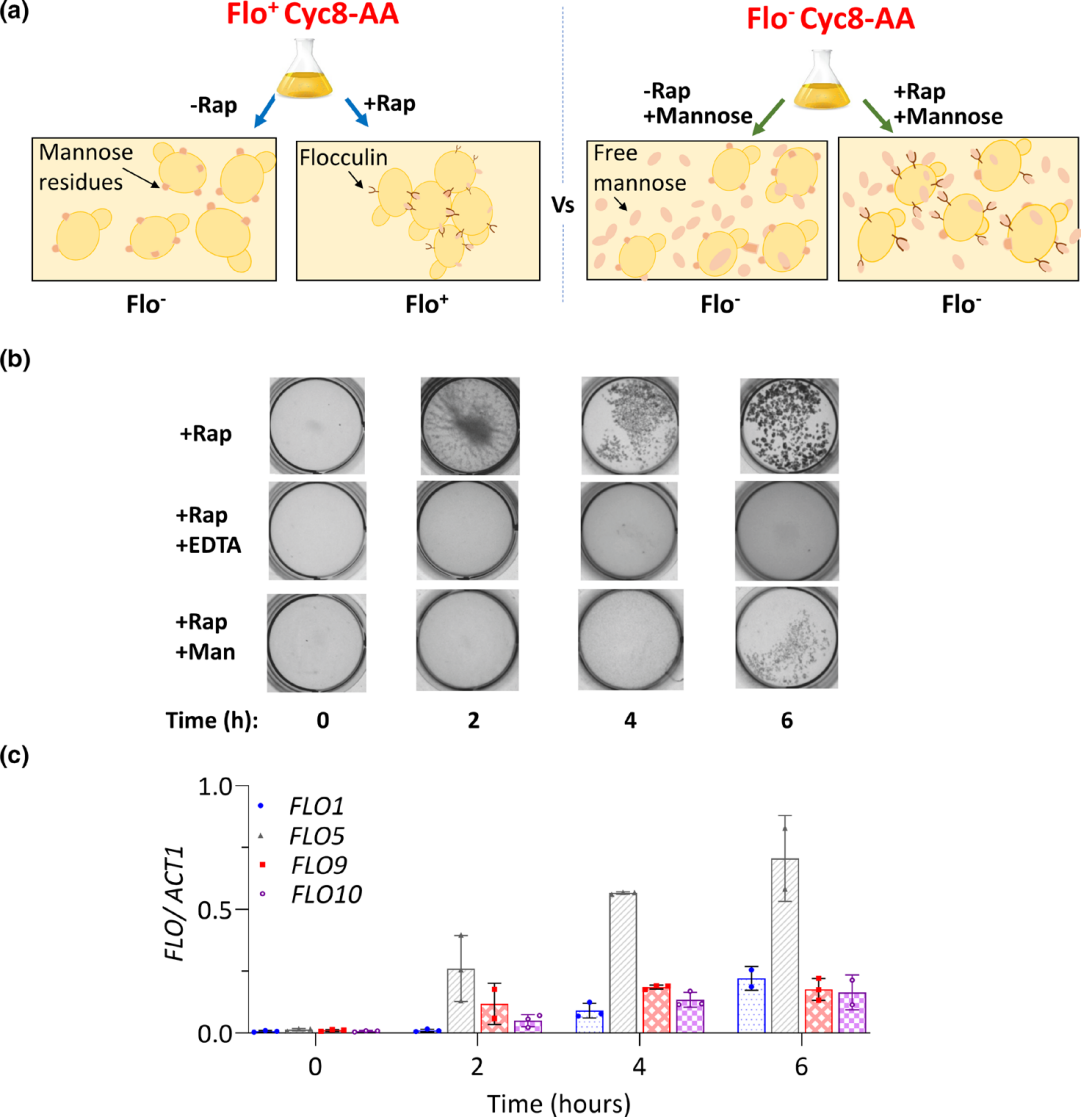
Assessing the impact of flocculation upon gene transcription following depletion of Cyc8p from the nucleus. (**a**) Experimental design to reveal the impact of flocculation upon global gene transcription in a Cyc8p conditional mutant. Transcriptome analysis was performed following rapamycin-induced Cyc8p depletion via the anchor-away technique (Cyc8-AA) in cells cultured in the absence of mannose, where flocculation was present (Flo^+^ Cyc8-AA), and in the presence of mannose, where flocculation was absent (Flo^−^ Cyc8-AA). The two transcriptomes were then compared. In the Flo^+^ Cyc8-AA experiment, a culture of Cyc8-AA cells was divided equally in two. Rapamycin (Rap) was added to one half of the culture and RNA-sequencing was performed on cells after 4 hours (h) of incubation (+Rap, Flo^+^). The other half of the culture was the control which was incubated for 4 h in the absence of rapamycin (-Rap, Flo^-^). In the Flo^-^ Cyc8-AA experiment, rapamycin and mannose (250 mM) were added to half the Cyc8-AA culture and RNA-sequencing was performed on cells after 4 h of incubation (+Rap, +Mannose, Flo^-^). The other half of the culture was the control which was incubated for 4 h in the absence of rapamycin and presence of mannose (-Rap, +Mannose, Flo^-^). (**b**) Images showing the presence or absence of the flocculation phenotype in Cyc8-AA cell cultures following growth after the addition of rapamycin in (**i**) the absence of EDTA and mannose (+Rap), (ii) the presence of EDTA (+Rap+EDTA) and (iii) the presence of mannose (+Rap+Man), for the times (hours, h) indicated. Time zero refers to immediately prior to rapamycin addition. (**c**) RT-qPCR analysis of mRNA levels of *FLO1*, *FLO5*, *FLO9* and *FLO10* from Cyc8-AA cells following the addition of rapamycin and depletion of Cyc8p from the nucleus. Time shown after rapamycin addition is indicated. Time zero refers to immediately prior to rapamycin addition. RT-qPCR values were normalized to *ACT1* mRNA, and error bars represent standard deviation from three biological replicates.

The anchor-away (AA) protocol was performed as previously described [35,57]. In both the Flo^+^ and Flo^-^ Cyc8-AA condition (see Fig. 1a) the Cyc8-AA strain was grown in YPD to early log phase (OD_600 nm_ of ~0.3) and the cultures were divided equally in two. In the Flo^+^ Cyc8-AA experiment, rapamycin (dissolved in DMSO) was added to the experimental half of the culture (+Rap), whereas DMSO alone was added to the control (-Rap) half of the culture. After 4 h of growth, RNA was extracted from both the control and experimental cultures. In the Flo^−^ Cyc8-AA experiment, rapamycin (dissolved in DMSO) and mannose were added to the experimental half of the culture (+Rap+Mannose), whereas mannose and DMSO were added to the control half (-Rap+Mannose). After 4 h of growth, RNA was prepared from both the control and experimental cultures. In each experiment, rapamycin (Fisher) was added at a final concentration of 1 µg ml^−1^ to initiate Cyc8p depletion, and mannose (VWR High Purity Grade, J443-500G) was added at a final concentration of 0.25 M to inhibit flocculation.

### RT-qPCR

RNA extraction, cDNA preparation and reverse transcription quantitative PCR (RT-qPCR) analysis were performed as previously described [4,35]. Values were normalized to *ACT1* RNA. Primers used are shown in Table S1, available in the online version of this article.

### RNA-sequencing

**Fig. 2.**
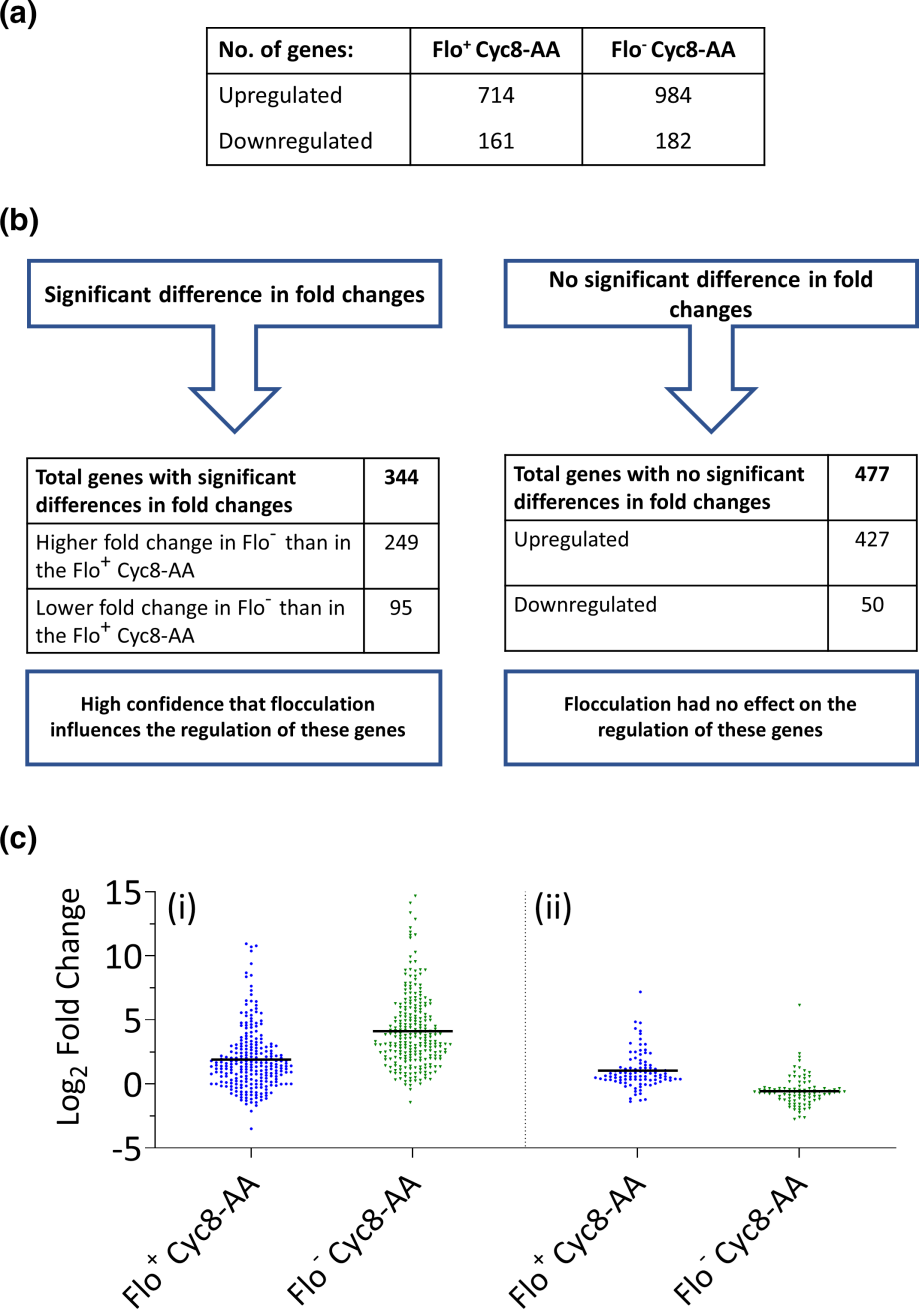
Comparison of the transcriptomes following Cyc8p depletion via anchor-away in flocculating (Flo^+^ Cyc8-AA) and non-flocculating (Flo^−^ Cyc8-AA) cells. (**a**) Table outlining the number of genes significantly up- and downregulated in the Flo^+^ and Flo^-^ Cyc8-AA experiments. (**b**) Schematic outlining the numbers of genes which showed a significant difference in their fold changes between the Flo^+^ and Flo^−^ Cyc8-AA experiments and which showed no significant differences in their fold changes between the Flo^+^ and Flo^−^ Cyc8-AA experiments. (**c**) Log_2_ fold change values of the 344 genes that showed a significant difference in their ratios between the Flo^+^ Cyc8-AA experiment compared to the Flo^−^ Cyc8-AA experiment separated into (**i**) the 249 genes that had significantly lower fold changes in the Flo^+^ Cyc8-AA experiment than in the Flo^−^ Cyc8-AA experiment, and (ii) the 95 genes that had significantly higher fold changes in the Flo^+^ Cyc8-AA experiment than in the Flo^−^ Cyc8-AA experiment.

**Fig. 3.**
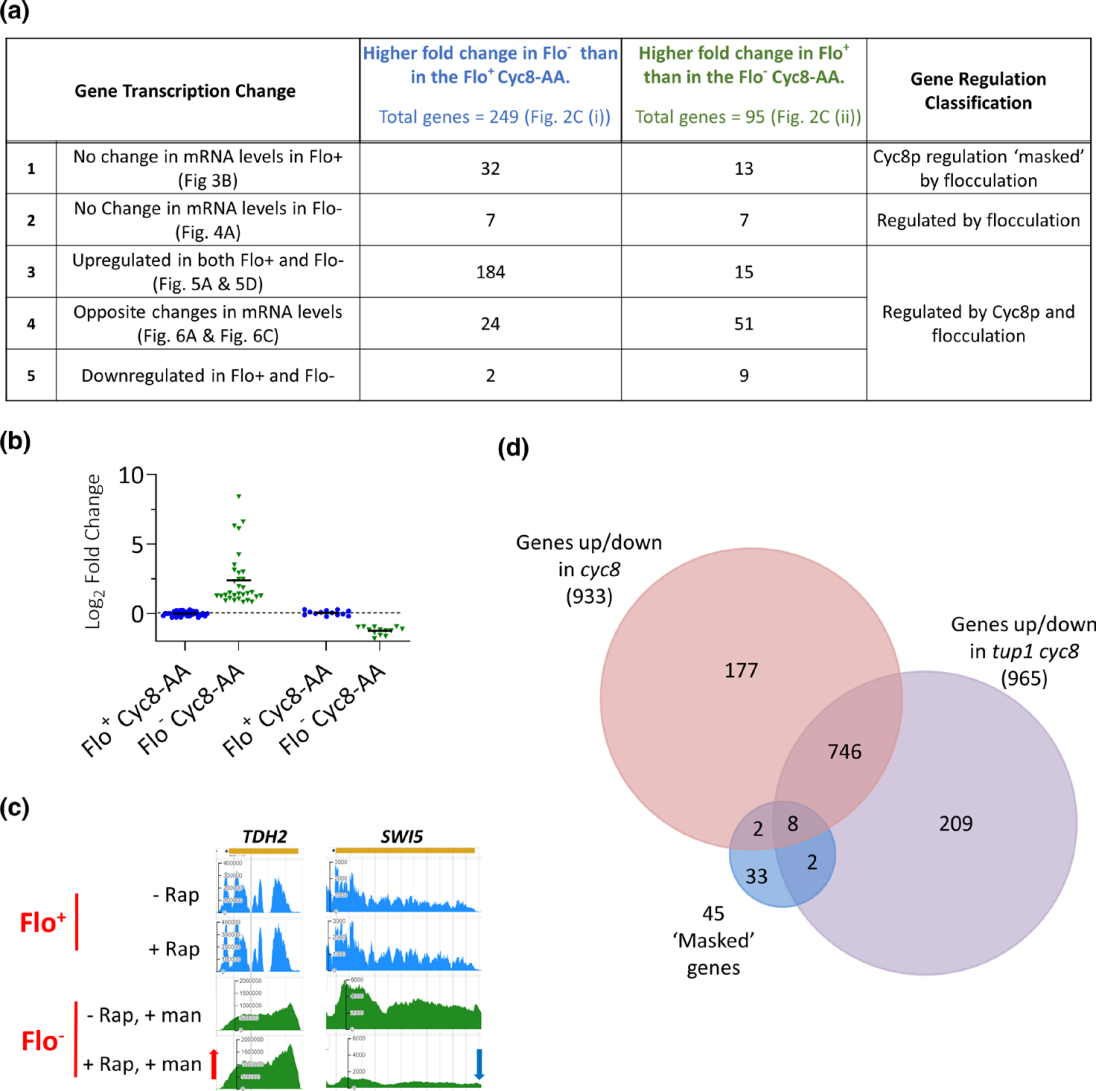
Cyc8p-regulated genes that are ‘masked’ by the flocculation phenotype. (**a**) Table summarizing the different ways that flocculation influences gene transcription. Row 1: mRNA levels of 45 genes that were unchanged in Flo^+^ Cyc8-AA cells, but showed changes (32 genes upregulated, 13 genes downregulated) in Flo^−^ Cyc8-AA cells. These genes were classified as being subject to Cyc8p regulation although the changes in transcription were masked by the flocculation phenotype. Row 2 : 14 genes showed changes in mRNA levels only in the presence of the flocculation phenotype; these genes were designated as being ‘regulated by flocculation’ only. Rows 3–5: genes regulated by both Cyc8p and flocculation. Row 3: 184 genes showed higher upregulation when flocculation was absent (Flo^−^ Cyc8-AA), and 15 genes showed higher upregulation when flocculation was present (Flo^+^ Cyc8-AA). Row 4: 75 genes showed opposite changes in mRNA levels depending on whether flocculation was present or absent. The figures depicting the different gene cohorts are shown in parentheses. (**b**) Log_2_ fold-changes of 45 genes that showed no significant change in mRNA levels in Flo^+^ Cyc8-AA cells but showed upregulation (32 genes) and downregulation (13 genes) in Flo^−^ Cyc8-AA cells. (**c**) JBrowse images of *TDH2* and *SWI5* mRNA levels measured by RNA-seq analysis. Red arrow represents upregulation, while blue arrow represents downregulation. (**d**) Venn diagram showing the overlap of the 45 genes which are regulated by Cyc8p, but whose changes in mRNA levels are masked by the flocculation phenotype, with genes significantly up- or downregulated in *cyc8* and *tup1 cyc8* gene deletion mutants.

**Fig. 4.**
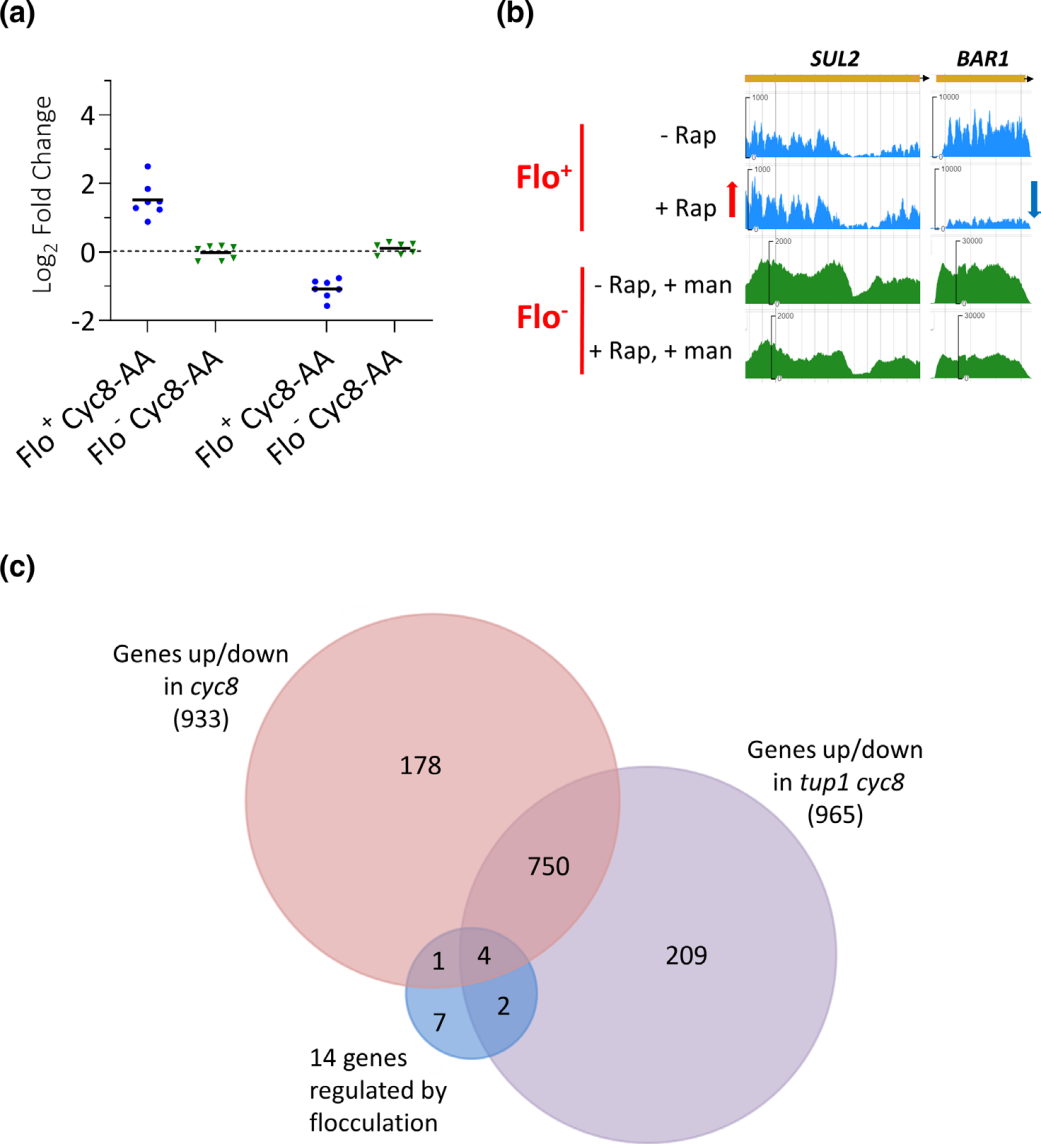
Genes specifically regulated by flocculation. (**a**) Log_2_ fold changes of the 14 genes that showed no significant change in mRNA levels in the Flo^−^ Cyc8-AA experiment but showed upregulation (seven genes) or downregulation (seven genes) in the Flo^+^ Cyc8-AA experiment. (**b**) JBrowse images of *SUL2* and *BAR1* mRNA levels measured by RNA-seq analysis. Red arrow represent upregulation, while blue arrow represents downregulation. (**c**) Venn diagram showing the overlap of the 14 genes regulated by the flocculation phenotype with genes significantly up- or downregulated in *cyc8* and *tup1 cyc8* gene deletion mutants.

**Fig. 5.**
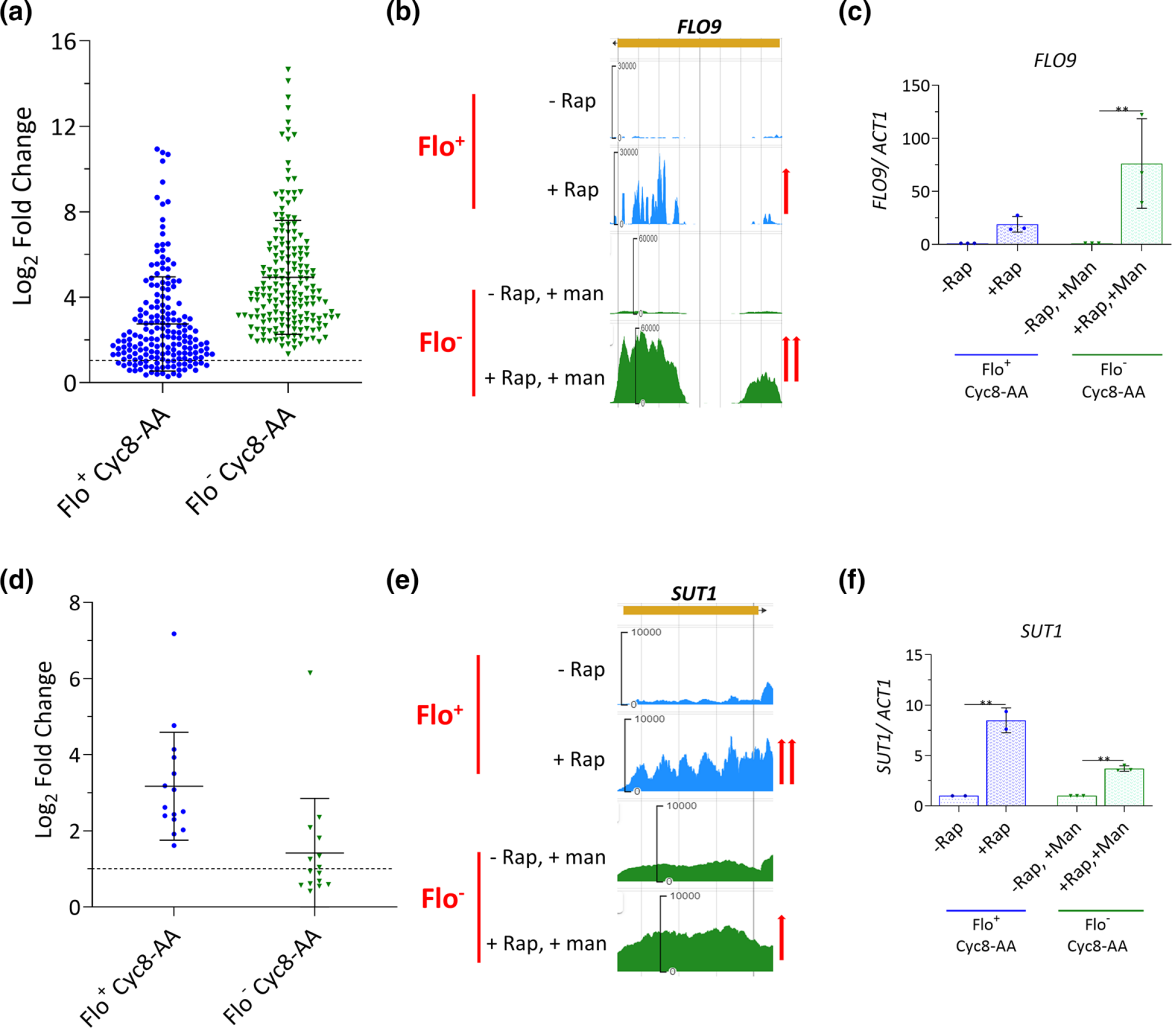
Genes regulated by Cyc8p and further influenced by flocculation. (**a**) Log_2_ fold changes of the 184 genes that showed upregulation in both the Flo^+^ Cyc8-AA and Flo^−^ Cyc8-AA experiments but showed higher upregulation in the non-flocculant (Flo^−^ Cyc8-AA) cells. (**b**) JBrowse images of *FLO9* mRNA levels measured by RNA-seq in Flo^+^ and Flo^-^ Cyc8-AA experiments; red arrows indicate the different extents of upregulation. (**c**) *FLO9* transcript levels measured using RT-qPCR in the Flo^+^ and Flo^−^ Cyc8-AA experiments. (**d**) Log_2_ fold changes of the 15 genes that showed upregulation in the Flo^+^ and Flo^−^ Cyc8-AA experiments but showed significantly lower upregulation in the non-flocculant (Flo^−^ Cyc8-AA) cells. (**e**) JBrowse images of mRNA levels of *SUT1* measured by RNA-seq; red arrows represent the different extents of upregulation. (**f**) *SUT1* transcript levels measured using RT-qPCR in Flo^+^ and Flo^−^ Cyc8-AA experiments. In (c) and (f), RT-qPCR values were normalized to *ACT1* mRNA. The mRNA values of each target gene after rapamycin addition (+Rap) were further normalized to the mRNA levels in the absence of rapamycin (-Rap), which were set at a value of 1. Error bars reflect standard deviation from three biological replicates (**P<0.005 determined by a one-way ANOVA).

**Fig. 6.**
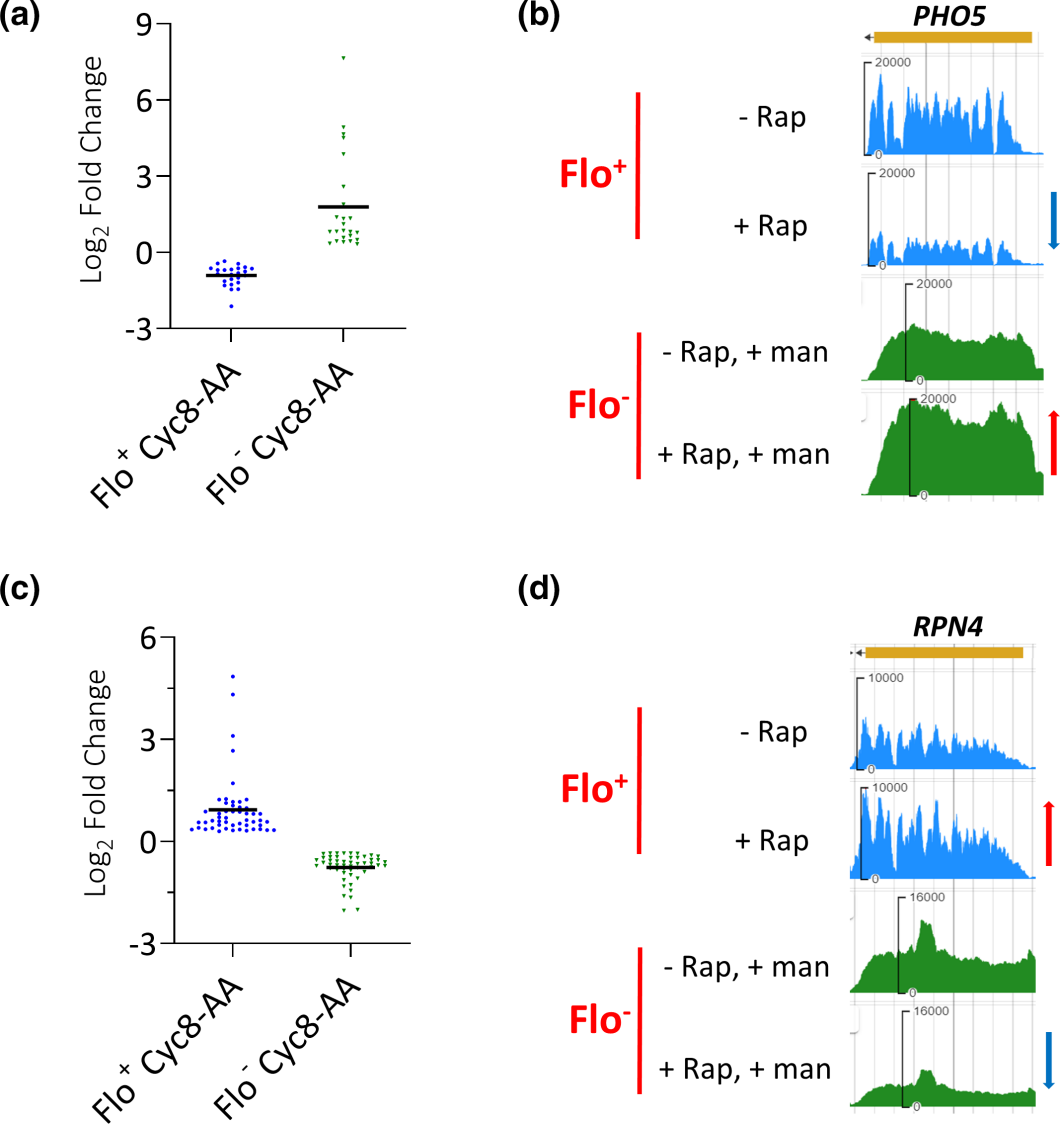
Genes showing opposite changes in mRNA levels in the presence or absence of the flocculation phenotype. (**a**) Log_2_ fold changes of the 24 genes that showed downregulation in the Flo^+^ Cyc8-AA experiment but upregulation in the Flo^−^ Cyc8-AA experiment. (**b**) JBrowse images of mRNA levels of *PHO5* measured by RNA-seq in the Flo^+^ and Flo^-^ Cyc8-AA experiments. (**c**) Log_2_ fold changes of the 51 genes that showed upregulation in Flo^+^ Cyc8-AA but downregulation in Flo^−^ Cyc8-AA. (**d**) JBrowse images of mRNA levels of *RPN4* measured by RNA-seq in Flo^+^ and Flo^−^ Cyc8-AA experiments. In all JBrowse images, the red arrow indicates upregulation, and the blue arrow represents downregulation.

**Fig. 7.**
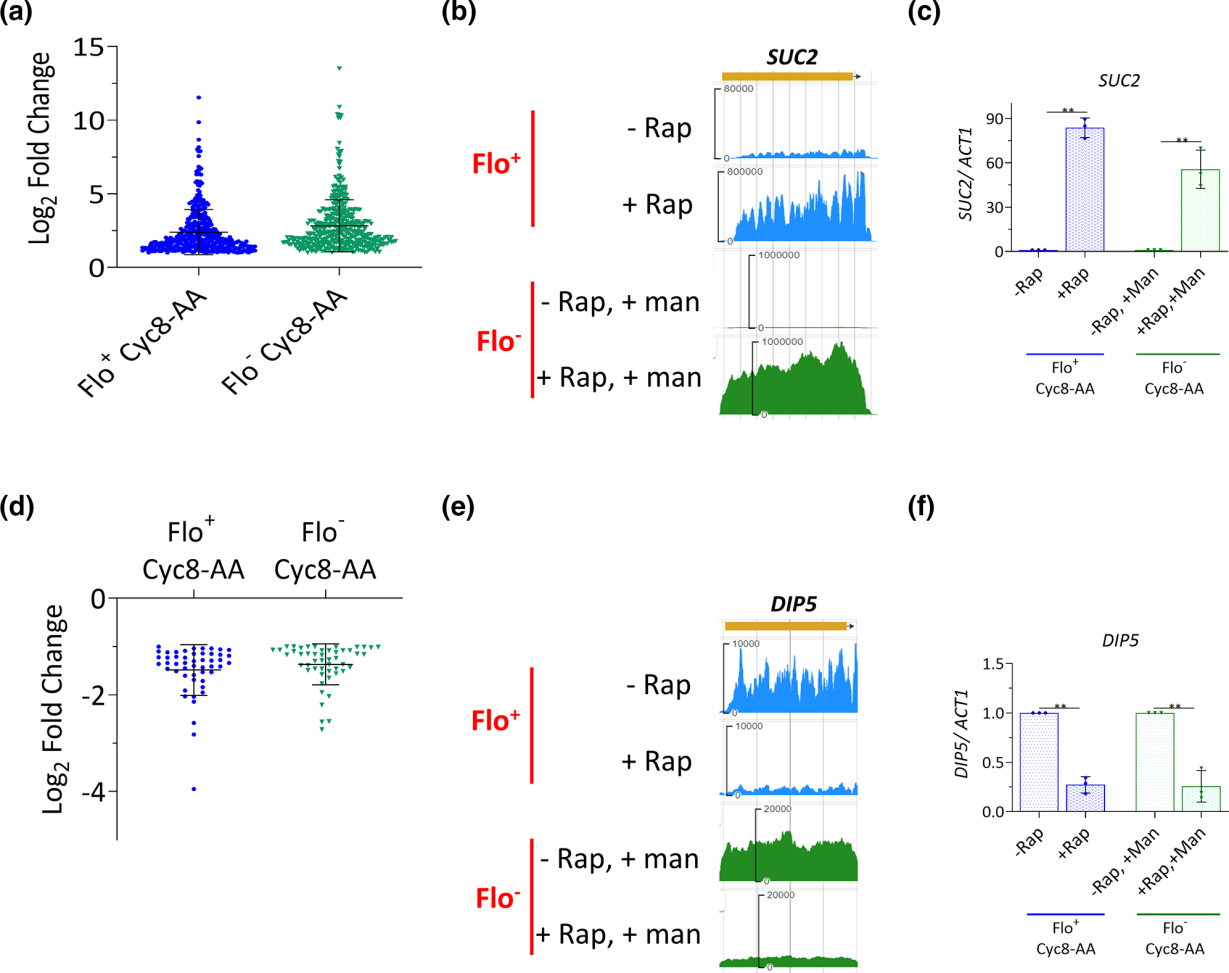
Cyc8p-regulated genes unaffected by flocculation. (**a**) Log_2_ fold change values of 427 genes at least twofold upregulated in both Flo^+^ and Flo^−^ Cyc8-AA experiments, with no significant difference in their ratios between the two experiments. (**b**) JBrowse images of mRNA levels of *SUC2* measured by RNA-seq in the Flo^+^ and Flo^−^ Cyc8-AA experiments. (**c**) *SUC2* transcript levels measured using RT-qPCR in the Flo^+^ and Flo^−^ Cyc8-AA experiments. (**d**) Log_2_ fold change values of 50 genes at least twofold downregulated in both the Flo^+^ and Flo^−^ Cyc8-AA experiments, with no significant difference in their ratios between the two experiments. (**e**) JBrowse images of mRNA levels of *DIP5* measured by RNA-seq in the Flo^+^ and Flo^−^ Cyc8-AA experiments. (**f**) *DIP5* transcript levels measured using RT-qPCR in Flo^+^ and Flo^−^ Cyc8-AA experiments. In (c) and (f), RT-qPCR values were normalized to *ACT1* mRNA and error bars reflect standard deviation from three biological replicates (**P<0.005 determined by a one-way ANOVA).

**Fig. 8.**
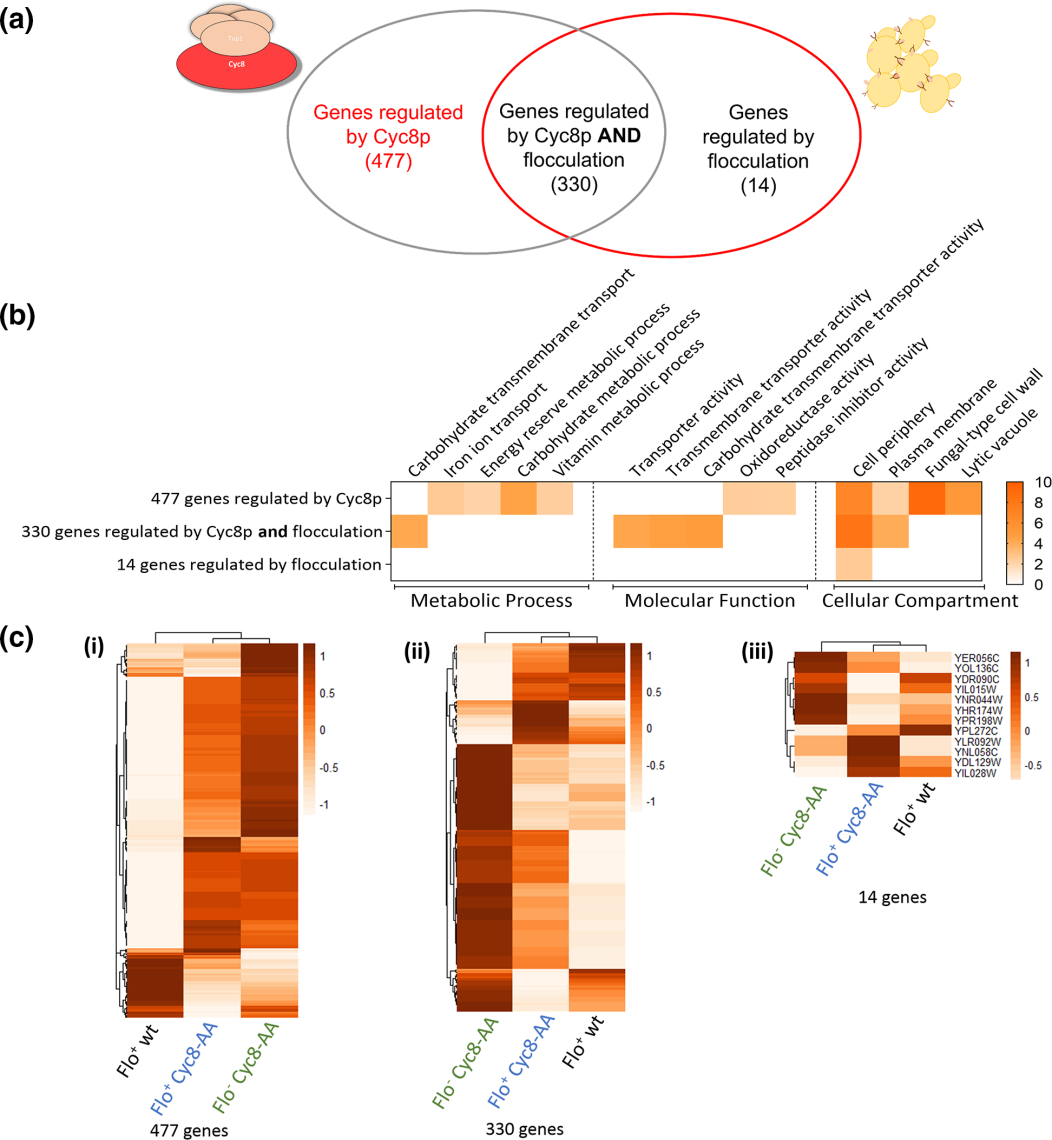
Comparison of genes solely regulated by Cyc8p or solely regulated by flocculation with genes regulated by Cyc8p and further influenced by flocculation. (**a**) Venn diagram illustrating the 477 genes regulated solely by Cyc8p, the 330 genes regulated by Cyc8p and flocculation, and the 14 genes regulated solely by flocculation. (**b**) Gene Ontology (GO) terms associated with the genes (**i**) regulated by Cyc8p only, (ii) regulated by Cyc8p and flocculation, and (iii) regulated solely by flocculation. (**c**) Heatmap of the log_2_ fold changes in a *FLO1* over-expression strain (*FLO1*^+^ wt) [27] compared to (**i**) the 477 genes regulated solely by Cyc8p, (ii) the 330 genes regulated by both Cyc8p and flocculation, and (iii) the 14 genes regulated solely by flocculation. The heatmap displays Z-scores for each gene; the legend can be interpreted as standard deviations above or below the mean fold change for each gene compared to the control condition with the dark orange colour indicating higher than average expression and paler orange colours representing lower than average expression of that gene. Each row represents a gene, and each column represents a different strain. The heatmap was generated using the pheatmap package in R-Studio which clusters genes together by their similarity in gene expression patterns.

RNA was extracted from exponentially growing cells using the Hot Phenol method and purified using the RNeasy Minelute Cleanup Kit (Qiagen) [4,58]. Total RNA was sent to Eurofins Genomics (Germany) for rRNA depletion, cDNA library preparation and strand-specific RNA sequencing (RNA-seq) using the Illumina Platform. Single-end sequencing was performed for the Flo^−^ Cyc8-AA experiment and paired-end sequencing was performed for the Flo^+^ Cyc8-AA experiment. Raw sequencing data were processed using fastp (v0.20.0) software to remove poor quality bases (below Phred Quality 20) or adapter sequences. High-quality reads were mapped to the *S. cerevisiae* S288c reference genome available on ENSEMBL using the STAR aligner (STAR v2.7.3) and BAM files were generated. Differential gene expression analysis using the DESeq2 Bioconductor package was carried out by Dr Karsten Hokamp (Trinity College Dublin). Using normalized counts, log_2_ fold changes and Benjamini–Hochberg adjusted *P*-values were then calculated. The Bigwig files, which represent coverage of mapped reads, were uploaded to JBrowse and are available to view at https://bioinf.gen.tcd.ie/jbrowse2/?config=AA_comparisons%2Fconfig.json. The RNA-seq datasets are available in the Gene Expression Omnibus (GEO) repository (GEO accession number GSE248550). The RNA-seq data used in Figs 2–8 are shown in Table S2.

### Comparison of ratios between the Flo^+^ and Flo^−^ Cyc8-AA datasets

The RNA datasets for the Flo^+^ Cyc8-AA (+Rap vs -Rap) and Flo^−^ Cyc8-AA (+Rap, +Man vs -Rap, +Man) comparison were generated at different times using different sequencing technology (single-end reads for the Flo^−^ Cyc8-AA experiment and paired-end reads for Flo^+^ Cyc8-AA experiment) which can introduce unwanted variation in the data due to technical differences and not due to true biological differences as a result of the presence or absence of the flocculation phenotype. Therefore, additional analysis using interaction terms in DESeq2, which compares the ratios between two RNA-seq datasets, was carried out [59]. Following this analysis, an adjusted *P*-value was calculated. For this study we set the parameters of |log_2_ fold change| ≥1 and an adjusted *P*-value of ≤0.01, as a significant difference in ratios between the two datasets.

### Chromatin immunoprecipitation (ChIP)

Locus-specific ChIP was performed as previously described [9,35]. Tup1p ChIP was carried out using 1.5 µl of a Tup1p antibody (a gift from J. Reese). Cyc8-FRB ChIP was performed using 5 µl of an anti-FRB antibody (20 mg ml^−1^) (Enzo Life Sciences, pAb.ALX-215-065-1). Occupancy signals were determined by comparing the enrichment of DNA found in the immunoprecipitated (IP) material versus the input (IN) material. The ChIP (IP/IN) signal at the *FLO1* promoter was then normalized to an IP/IN signal at an internal negative control region (*Int-V*) to give ‘relative occupancy’, where indicated (Fig. S4). Primers used are shown in Table S1.

### Statistical software

Statistical analysis was carried out using GraphPad Prism 8. To assess the statistical significance of a result an ANOVA was carried out. Venn diagrams were constructed using Funrich software. Heatmaps were generated using the pheatmap function in R-Studio. The heatmap displays Z-scores for each gene; the legend can be interpreted as standard deviations above or below the mean fold change for each gene compared to the wild-type, with the dark orange colour indicating higher than average expression and paler orange colours representing lower than average expression of that gene. Each row represents a gene, and each column represents an experimental condition, as described accordingly.

### Gene Ontology analysis

Using the Gene Ontology Term Finder (v0.86) the significant (*P*<0.01) GO terms were retrieved from the Saccharomyces Genome Database (SGD) website [60]. The terms were divided into three sub-ontologies: Metabolic Process describes broad biological processes, Molecular Function refers to basic activities of the gene products and Cellular Component refers to location of the gene product. The heatmap was generated using GraphPad Prism 8, the scale represents the −log_10_ (corrected *P*-value), and higher values indicate greater statistical significance. The heatmap in Fig. 8b shows representative GO terms from each category. The complete list of all significant GO terms and the list of genes associated with each term is available in Table S3) .

## Results

Most previous studies to identify the genes under the control of the Tup1-Cyc8 complex have come from the transcriptomes of *TUP1* and/or *CYC8* deletion or conditional mutants which exhibit strong flocculation phenotypes [3,4, 14]. We hypothesized that the *tup1* and *cyc8* mutant cells in the flocs would be subject to nutrient and oxygen limitation which would influence the resultant transcriptomes. If correct, this would mean all previous *cyc8* and *tup1* transcriptomes would contain genes subject to *CYC8* and *TUP1* regulation, and genes indirectly influenced by flocculation.

In this study, we have uncovered the impact of flocculation upon the transcription profile of a Cyc8p-deficient strain by comparing the transcriptomes of a Cyc8p anchor-away (Cyc8-AA) strain in the presence and absence of flocculation (Fig. 1a) [57]. Anchor-away is a technique by which a protein of interest, in this case Cyc8p, can be rapidly removed from the nucleus following the addition of rapamycin to the appropriately constructed strain. Cyc8p was targeted for depletion, and not Tup1p, because the Cyc8p-depleted strain showed the greatest flocculation phenotype compared to the Tup1p depletion strain and expressed the *FLO* genes to the greatest extent (Figs S1 and S2). The strain background used for the anchor-away technique has been engineered to be insensitive to rapamycin. Thus, in this study, although the addition of rapamycin induces loss of Cyc8p from the nucleus, there are no deleterious effects of rapamycin directly upon cell function.

First, we determined the transcriptome of a Cyc8-AA strain grown under conditions in which flocculation was present (Fig. 1a; Flo^+^ Cyc8-AA). We then repeated the analysis of Cyc8-AA cells grown in the presence of mannose which abolishes flocculation (Fig. 1a; Flo^−^ Cyc8-AA). We then compared the two transcriptomes to uncover the impact of flocculation upon global transcription in the absence of Cyc8p (Fig. 1a; Flo^−^ Cyc8-AA vs Flo^+^ Cyc8-AA). Importantly, the transcription profile of the mannose-treated, non-flocculating Cyc8p-depleted cells [Fig. 1a; Flo^−^ Cyc8-AA: +Rap, +Man (Flo^−^)] was normalized to the transcriptome of mannose-treated cells in which Cyc8p was not depleted [Fig. 1a; Flo^-^ Cyc8-AA: -Rap, +Man (Flo^−^)]. Thus, we propose that mannose-dependent changes in gene transcription (Table S4) would have been controlled for in the mannose-treated experiment (Flo^−^ Cyc8-AA) and excluded from the subsequent Flo^−^ Cyc8-AA versus Flo^+^ Cyc8-AA comparison (Fig. 1a).

### Flocculation is abolished in the presence of mannose

To minimize the impact of mannose upon cells, we wanted to use the lowest concentration of mannose to inhibit flocculation, and to expose cells to this mannose for the shortest possible time during the Cyc8p anchor-away. Prior to performing the transcriptome comparison, we therefore first established the minimum amount of mannose which would inhibit flocculation following anchor-away of Cyc8p (Fig. S3). We also confirmed that rapamycin-induced depletion of Cyc8p from the nucleus in the anchor-away strain was unaffected by the addition of mannose (Fig. S4).

We next performed a time-course analysis of flocculation and *FLO* gene transcription following rapamycin-induced Cyc8p depletion (Fig. 1b, c). In the absence of rapamycin, no flocculation or *FLO* gene transcription was detected, consistent with Tup1-Cyc8-dependent repression of the *FLO* genes [Fig. 1b, c (+Rap, 0 h)] [29]. At 2 h after rapamycin addition, flocculation was visible and *FLO5*, *9* and *10* gene transcription could be detected [Fig. 1b, c (+Rap, 2 h)]. At 4 h after rapamycin addition, increased transcription of all four *FLO* genes was detected and cells could be seen to form larger and more visible flocs than those seen at the 2 h time point. At 6 h after rapamycin addition, although flocculation was more visible than before, there was no significant increase in *FLO* gene transcription compared to that detected at 4 h post-rapamycin addition.

The addition of EDTA to the rapamycin-treated cells at each time point dispersed the flocs, confirming the cell aggregation observed was a consequence of flocculation (Fig. 1b, +Rap+EDTA).

Addition of mannose to cell cultures at a final concentration of 250 mM immediately prior to rapamycin addition prevented flocculation at the 2 and 4 h post-rapamycin time points, although some flocculation was visible at the 6 h time point (Fig. 1b, +Rap+Man). The flocculation visible at the 6 h time point was probably due to depletion of mannose from the medium to a level below that needed to out-compete flocculin binding [61].

We therefore chose the 4 h post-rapamycin time point to perform the Cyc8-AA RNA-seq analysis in the presence and absence of flocculation (Fig. 1a). At this time point, the data suggest that *FLO* gene transcription has plateaued, and mannose has prevented floc formation. Importantly, we confirm the finding from previous studies to show that both Cyc8p and Tup1p are removed from the *FLO1* gene 30 min after rapamycin addition in the Cyc8-AA strain, and that Cyc8p (and Tup1p) depletion persists over the time period used here (Fig. S4) [35,57]. Thus, at the 4 h time point after rapamycin addition in the presence or absence of mannose, these data suggest that Tup1-Cyc8-dependent gene regulation should be fully abolished.

### RNA-seq analysis in flocculating and non-flocculating Cyc8p-depleted cells

To assess the impact of flocculation upon the transcriptome of a Cyc8p-deficient strain, the transcriptomes of a flocculating (Flo^+^) and non-flocculating (Flo^−^) Cyc8-AA experiment were compared (Fig. 1a; Flo^+^ Cyc8-AA vs Flo^−^ Cyc8-AA). The difference between the Flo^+^ and Flo^−^ Cyc8-AA transcriptomes was then analysed to reveal the impact of flocculation upon global transcription in cells following the depletion of Cyc8p from the nucleus.

The results of RNA-seq analysis following Cyc8p anchor-away in flocculating cells [Fig. 1a; Flo^+^ Cyc8-AA: +Rap (Flo^+^) vs -Rap (Flo^−^)] revealed 714 genes were upregulated and 161 genes were downregulated (Fig. 2a). Following RNA-seq analysis of the Cyc8p anchor-away strain grown under the non-flocculating conditions [Fig. 1a; Flo^−^ Cyc8-AA: +Rap, +Man (Flo^−^) vs -Rap, +Man (Flo^−^)], 984 genes were upregulated and 182 genes were downregulated (Fig. 2a).

To compare the resultant Flo^+^ Cyc8-AA and Flo^−^ Cyc8-AA RNA-seq datasets described above, analysis using interaction terms was performed [59]. This analysis compares the ratios of fold changes in the flocculating Flo^+^ Cyc8-AA (+Rap vs -Rap) experiment to the ratios in the non-flocculating Flo^−^ Cyc8-AA (+Rap, +Man vs -Rap, +Man) experiment, to yield interaction terms.

The analysis revealed that 344 genes had a significant difference in their ratios between the two experiments which suggests that these genes were influenced by the flocculation phenotype (Fig. 2b). Of these genes, 249 showed higher fold changes in the Flo^−^ Cyc8-AA experiment compared to the Flo^+^ Cyc8-AA experiment suggesting that flocculation had a negative effect on transcription of these genes [Fig. 2c(i)]. Conversely, 95 of the 344 genes showed lower fold changes in the Flo^−^ Cyc8-AA experiment compared to the Flo^+^ Cyc8-AA experiment suggesting that flocculation had a positive effect on the transcription of these genes [Fig. 2c(ii)]. Together, this suggests that when flocculation did influence gene transcription, this phenotype predominantly had a negative effect.

The results also showed a group of 477 genes that were up- or downregulated in both the Flo^+^ and Flo^−^ Cyc8-AA experiments which had no significant difference in their fold changes between the two experiments (Fig. 2b). This indicates that the flocculation phenotype had no effect on the mRNA levels of this set of Cyc8p-dependent genes.

### Identification of Cyc8p-regulated genes masked by the flocculation phenotype

Within the 344 genes influenced by flocculation, a group of 45 genes showed no significant changes in mRNA levels in the flocculant (Flo^+^) Cyc8-AA cells, but were up- or downregulated in the non-flocculant (Flo^−^) Cyc8p-depleted cells (Fig. 3a, row 1). This result suggests that although this set of 45 genes are regulated by the Tup1-Cyc8 complex, the presence of the flocculant phenotype in the *cyc8* mutant negates the *CYC8*-dependent changes in their mRNA levels. Within this set of 45 genes, 32 genes showed upregulation and 13 genes showed downregulation solely in the non-flocculant Cyc8p-depleted cells (Fig. 3b, see Flo^−^ Cyc8-AA data).

Examples of genes that are subject to negative and positive regulation by Cyc8p but whose transcription changes were masked by flocculation were *TDH2*, which encodes glyceraldehyde-3-phosphate dehydrogenase, and *SWI5*, which encodes a transcription factor, respectively (Fig. 3c, compare 'Flo^−^, +Rap, +man' to 'Flo^−^, -Rap, +man'; Fig. S5a) [62,63]. The difference in coverage between the mapped reads shown in the JBrowse images for the Flo^+^ (blue traces) and Flo^−^ AA experiments (green traces) is due to the different type of reads (paired-end vs single-end, respectively) that were used to generate the two RNA-seq data sets (see Methods).

These data suggest that these genes would have been missed from previously published gene transcription profiles from *cyc8* deletion mutants as they would have been masked due to the impact of the flocculation in these strains. To test this, we compared these ‘masked’ genes with a recently published transcriptome from a flocculant *cyc8* deletion mutant and confirmed that 35 of the 45 ‘masked’ genes were not detected as being differentially expressed in the *cyc8* mutant (Fig. 3d, compare 45 ‘masked’ genes with genes up- and downregulated in *cyc8*) [4].

### A Cyc8-AA strain might be more similar to a *tup1 cyc8* double deletion mutant than a *cyc8* single mutant

Consistent with previously published data, our gene-specific validation of the Cyc8-AA at the *FLO1* gene showed that after rapamycin addition, Tup1p was also lost from the *FLO1* promoter (Fig. S4b, c) [35]. This suggests that removal of Cyc8p via anchor-away also causes the removal of Tup1p from the *FLO1* gene. If Tup1p loss is occurring at all Tup1-Cyc8-regulated genes following Cyc8p depletion, it would suggest that a Cyc8-AA strain would be more similar to a *tup1 cyc8* double deletion mutant than a *cyc8* single deletion mutant. We therefore compared the 45 newly identified Cyc8p-dependent genes masked by flocculation with the recently published transcriptome of a *tup1 cyc8* double deletion mutant to determine if the 45 masked genes were also absent from the *tup1 cyc8* transcriptome (Fig. 3d, compare 45 ‘masked genes’ with genes up- and downregulated in *tup1 cyc8*) [4]. The data again showed that 35 out of the 45 masked genes were not detected as being differentially expressed in the *tup1 cyc8* double deletion mutant. Together, this suggests that our analysis has identified 33 new genes subject to Tup1-Cyc8-dependent regulation that were missed from at least one previous Tup1-Cyc8-dependent gene transcription profile having been masked by the flocculation phenotype of the *cyc8* and *tup1* mutant strains analysed.

### Identification of genes solely regulated by flocculation in Cyc8p-depleted cells

Our data revealed a group of 14 genes that were up- or downregulated only in the flocculant Flo^+^ Cyc8-AA strain but showed no significant changes in mRNA levels in the non-flocculant Flo^−^ Cyc8-AA cells (Fig. 3a, row 2, and Fig. 4a). This suggests that the changes in mRNA levels of these genes were due solely to the presence of the flocculation phenotype, rather than the loss of Cyc8p from the nucleus. Interestingly, seven of the 14 genes were upregulated and seven were downregulated in the Flo^+^ Cyc8-AA strain (Fig. 4a). Examples of these flocculation-dependent up- and downregulated genes were *SUL2*, which encodes a sulphate permease, and *BAR1,* which encodes a protease involved in mating, respectively (Fig. 4b, compare 'Flo^+^, +Rap' to 'Flo^+^, -Rap'; Fig. S5b) [64,65]. These data suggest that this set of 14 genes will probably have been misinterpreted as being regulated by the Tup1-Cyc8 complex in previously published transcriptomes generated from flocculating *tup1* and *cyc8* mutants. Indeed, when the 14 flocculation-dependent genes were compared to the transcriptome of a *cyc8* single and *tup1 cyc8* double deletion mutant, seven genes were found to be included as being differential expressed within these transcriptomes (Fig. 4c) [4]. Thus, these seven genes can be excluded as being regulated by the Tup1-Cyc8 complex since their altered transcription in the absence of Cyc8p is due to flocculation and not the loss of Cyc8p.

### Genes regulated by Cyc8p and further influenced by flocculation

We next examined genes that were upregulated in both the Flo^+^ Cyc8-AA and Flo^−^ Cyc8-AA experiments which had significant differences in their fold changes compared to each other. A group of 184 genes showed significantly higher fold changes in the Flo^−^ cells compared to the Flo^+^ Cyc8-AA cells (Fig. 3a, row 3, and Fig. 5a). This result shows that this set of 184 genes were upregulated due to the removal of Cyc8p from the nucleus and were further upregulated in the absence of the flocculant phenotype. Thus, flocculation had a repressive effect on the transcription of this set of genes in the flocculant Flo^+^ Cyc8-AA strain. Although genes that showed modest upregulation (log_2_ fold change ≥0.3) in the Flo^+^ cells were included in this cohort, most of these genes (~74 %) were more than twofold upregulated in both Flo^+^ and Flo^-^ Cyc8-AA conditions compared to the relevant controls. Thus, flocculation limits de-repression of these genes in the absence of Cyc8p. Interestingly, an example of one of these genes was *FLO9* suggesting a possible feedback mechanism of flocculation to reduce *FLO* gene expression and limit flocculation (Fig. 5b, c).

Conversely, the results revealed 15 genes which showed upregulation in both the Flo^+^ and Flo^−^ Cyc8-AA experiments that had significantly higher fold changes when flocculation was present (Flo^+^ Cyc8-AA) (Fig. 3a, row 3, and Fig. 5d). This indicates that this set of 15 genes were upregulated due to the removal of Cyc8p from the nucleus and were further upregulated in the presence of flocculation. This suggests that flocculation had a positive role in the regulation of this set of genes. An example of one of these genes was *SUT1* (Fig. 5e, f) which encodes a transcription factor which positively regulates genes involved in sterol uptake under anaerobic conditions [66]. Thus, flocculation is acting to reinforce transcription of these genes in the absence of Cyc8p.

### Cyc8p-dependent genes subject to antagonistic regulation by flocculation

Further analysis of the group of 249 genes showing a greater fold change in transcription in the Flo^−^ Cyc8-AA experiment compared to that in the Flo^+^ Cyc8-AA experiment [see Fig. 2c(i)] revealed 24 genes that showed opposite changes in mRNA levels in response to the presence or absence of flocculation (Fig. 3a, row 4, and Fig. 6a). Surprisingly, these genes were upregulated in the non-flocculant Flo^−^ Cyc8-AA cells and downregulated in the flocculant Flo^+^ Cyc8-AA strain. Thus, these Cyc8p-dependent genes were negatively regulated by the presence of flocculation but were positively influenced when flocculation was absent. An example of a gene showing this behaviour was *PHO5* which encodes a secreted acid phosphatase that is induced under low-phosphate conditions and repressed when phosphate levels are high (Figs 6b and S5c) [67].

Similarly, within the group of 95 genes that had a greater fold change in transcription in the Flo^+^ Cyc8-AA experiment compared to that in the Flo^−^ Cyc8-AA experiment [see Fig. 2c(ii)], a cohort of 51 genes showed upregulation in flocculating (Flo^+^) cells and downregulation in non-flocculating (Flo^−^) cells (Fig. 3a, row 4, and Fig. 6c). Thus, these Cyc8p-regulated genes are positively influenced in the presence of flocculation and negatively influenced when there is no flocculation. An example of a gene showing this behaviour was *RPN4* which encodes a stress responsive transcription factor that promotes expression of proteasome genes (Fig. 6d) [68].

Together, these results reveal that some Cyc8p-dependent genes can be antagonistically regulated depending upon the presence or absence of flocculation.

### Cyc8p-regulated genes unaffected by flocculation

Comparison of the transcriptomes of the flocculating Cyc8-AA strain and the non-flocculating Cyc8-AA strain revealed a group of 427 genes that were commonly upregulated in both the Flo^+^ and the Flo^−^ datasets (Fig. 7) (Figs2b  7a). This indicates that the presence or absence of flocculation did not affect the transcription of these Cyc8p-dependent genes. Instead, these genes were upregulated solely due to the conditional removal of Cyc8p from the nucleus and represent those genes at which Cyc8p is acting as a repressor of transcription. An example of one of these genes was *SUC2* which encodes the glucose-repressed invertase enzyme (Fig. 7b, c) [69].

The data also revealed a group of 50 genes that were at least twofold downregulated in both Flo^+^ Cyc8-AA and Flo^−^ Cyc8-AA conditions with no significant differences in the fold changes between the two datasets (Figs2b  7d). This indicates that this group of genes were downregulated solely due to the removal of Cyc8p from the nucleus and represents those genes at which Cyc8p is functioning as an activator of transcription. An example of one of these genes was *DIP5* which encodes a permease involved in amino acid uptake (Fig. 7e, f) [70].

### GO analysis of Cyc8p-dependent genes, Cyc8p-dependent genes further influenced by flocculation, and flocculation-dependent genes

We have uncovered (i) 477 genes subject solely to Cyc8p-dependent regulation, (ii) 330 Cyc8p-regulated genes that are further influenced by flocculation and (iii) 14 genes subject solely to regulation by flocculation (Fig. 8a). GO analysis of the genes within the three differently regulated cohorts revealed significant differences (Fig. 8b).

Consistent with published data, we found that the unique Cyc8p-regulated genes (477) were enriched for genes involved in carbohydrate metabolic processes and the fungal cell wall [2,3]. However, the 330 genes subject to Cyc8p regulation that were further influenced by flocculation were enriched for distinct categories including genes involved in sugar transport. Furthermore, the 14 genes solely regulated by flocculation were enriched for those involved in the cell periphery.

To further investigate the three gene cohorts identified in our study, we compared the three distinct transcription profiles we had identified with transcription data from a study where the *FLO1* protein was over-expressed in a non-flocculant wild-type (wt) strain to generate a strain showing a hyper-flocculation phenotype [27]. The authors of this study used a *GAL*-driven *FLO1* gene to over-express Flo1p and found that their hyper-flocculant strain had a transcription profile distinct from the non-flocculant wt strain. We therefore predicted that there would be significant overlap of the gene cohorts we had shown were subject to influence by flocculation with the genes identified as being uniquely regulated by flocculation in the *FLO1* over-expression study.

The results shown in the heat maps revealed that this was indeed the case (Fig. 8c). Comparison of the 477 Cyc8p-dependent genes not influenced by flocculation with the *FLO1* over-expression data showed that mRNA levels of these genes in the two Cyc8-AA experiments were more similar to each other than to the *FLO1* over-expression data [Fig. 8c(i)]. Conversely, the 330 Cyc8p-dependent genes which were further influenced by flocculation, and the 14 genes subject solely to regulation by flocculation, showed more similarity with the *FLO1* over-expression data than with the Flo^−^ Cyc8-AA read-out [Fig. 8c(ii and iii)]. Thus, both our study and the Flo1p over-expression study revealed a striking, and potentially similar, impact of flocculation upon global gene transcription of cells found within flocs.

## Discussion

In this study we investigated the impact of flocculation upon global transcription in a Cyc8p conditional mutant. The results revealed that of the 875 genes up- and downregulated in the absence of Cyc8p, 54 % were subject solely to Cyc8p-dependent regulation, 38 % were regulated by Cyc8p and further influenced by flocculation, and just 0.014 % were regulated by flocculation only and not by Cyc8p (Fig. 8a). This yielded an improved list of Cyc8p-regulated genes that includes newly identified Cyc8p-regulated genes that were masked by the flocculation phenotype and excludes the genes that were indirectly influenced by flocculation and were not regulated by Cyc8p. Furthermore, our analysis revealed that flocculation exerts a widespread and complex influence upon global gene transcription in the absence of Cyc8p whereby many Cyc8p-regulated genes can be either positively or negatively impacted depending upon the presence or absence of the flocculation phenotype.

Indeed, we found genes subject to negative and positive regulation by Cyc8p, but whose transcription changes were masked by the impact of flocculation (Fig. 3b, c). This means that the flocculation phenotype negates the transcription fold change caused due to the loss of Cyc8p and thereby masked these genes from having previously been recognized as being *CYC8*-dependent genes. Our analysis therefore updates the Cyc8p transcriptome to include 35 of these genes which we confirm were missed from at least one recent transcriptome obtained using *cyc8* deletion mutant cells [4].

Included within this set of newly identified Cyc8p-dependent genes is the *SWI5* gene which our data suggest is positively regulated by Cyc8p, but whose transcription fold change in a *cyc8* mutant is masked by the flocculation phenotype (Fig. 3c). Since Swi5p is a key transcription factor predominantly associated with activation of transcription, this supports a role for Tup1-Cyc8 in activating transcription, a least under conditions when flocculation is absent or weak [71].

We also show Cyc8p repressed genes that can be further repressed by flocculation and genes subject to positive regulation by Cyc8p whose transcription is enhanced by flocculation (Fig. 5). For example, the *FLO9* gene, which encodes a flocculin protein and contributes to flocculation, is repressed by Cyc8p in wt cells [29]. However, following *FLO9* de-repression in the absence of Cyc8p, *FLO9* transcription is then subject to negative regulation by the subsequent flocculation phenotype (Fig. 5b, c). Thus, flocculation is acting to limit transcription of at least one gene that contributes to the flocculation phenotype itself. It is tempting to speculate that this negative feedback of flocculation to *FLO9* transcription could help mitigate the potential deleterious impacts of flocculation upon the cell, such as reducing oxygen and nutrient availability, so that their effects do not become too harmful. This feedback could therefore act to ensure the optimal level of flocculation is achieved once the cells are triggered to embark on what should be a survival strategy and not a cause of further stress. Indeed, this regulation could act to balance the cost–benefit ratio of the flocculation survival strategy [72,73].

Conversely, flocculation is acting to reinforce transcription of the *SUT1* gene which our data show is subject to positive regulation of transcription by Cyc8p (Fig. 5d, f). Interestingly, the *SUT1* gene is known to be induced under conditions of low oxygen and encodes a transcription factor purported to physically interact with Cyc8p to then activate sterol uptake genes under anaerobic conditions [74]. Our data therefore show that Cyc8p is involved in activation of transcription of this gene, and that flocculation then reinforces transcription of this gene in the absence of Cyc8p. Thus, cooperation between Cyc8p and flocculation could be contributing to the dynamic transcriptional cell response to enable optimal adaptation to changes in oxygen availability as the flocculation phenotype develops.

When we further examined the 249 genes in which the fold change in transcription following Cyc8p depletion was greatest in the non-flocculant cells versus what was seen when flocculation was present, we found a cohort of 24 genes that were differentially regulated according to the presence or absence of flocculation (Fig. 3a, row 4). Surprisingly at these genes, as illustrated by *PHO5*, in the presence of flocculation, the loss of Cyc8p resulted in downregulation of transcription suggesting Cyc8p was negatively regulating *PHO5* transcription in the wt (Fig. 5b, Flo^+^, +Rap, blue arrow). Conversely, when the Cyc8p depletion was performed in the absence of flocculation, the gene was upregulated indicating that under these conditions Cyc8p was initially acting as a repressor (Fig. 5b, Flo^−^, +Rap+Man, red arrow). This intriguing result suggests that flocculation can determine whether Cyc8p has a positive or negative role upon transcription of some target genes.

A similar result was seen within the group of 95 genes that had a greater fold change in transcription in the Flo^+^ AA experiment compared to that in the Flo^−^ AA experiment. In this case, 51 genes showed upregulation in flocculating (Flo^+^) cells and downregulation in non-flocculating (Flo^−^) cells (Fig. 3, row 4, and Fig. 6c). Thus, these genes are positively influenced in the presence of flocculation and negatively influenced when there is no flocculation.

These data illustrate the complex influence that flocculation can have upon gene transcription. However, more work will need to be done to understand (i) why specific cohorts of genes are subject to the different types of regulation by flocculation, (ii) what the mechanism is that governs this regulation and (iii) what the consequence to cell function is of this flocculation-dependent regulation.

The starting hypothesis of this study was that flocculation would be indirectly impacting a *cyc8* transcriptome by influencing genes involved in oxygen and nutrient depletion caused by the aggregation of cells in the flocs. We predicted that the *cyc8* transcriptome would therefore contain numerous genes involved in the response to nutrient and oxygen depletion that would not be directly Cyc8p dependent. Surprisingly, we found only 14 genes which were shown to be influenced solely by flocculation and not by Cyc8p. Instead, our data revealed that flocculation had a much more widespread and varied influence upon global gene transcription, as opposed to flocculation exerting an absolute switching off or on of genes.

We chose to use a conditional Cyc8p mutant for this study, as opposed to a *cyc8* deletion mutant, for the following reasons. First, this was to minimize secondary impacts upon transcription and cell function that can occur following prolonged growth of deletion mutants [75]. Second, by using the conditional mutant, we could analyse the impact of flocculation upon transcription following exposure of cells to this phenotype over a minimal time (4 h). Third, by adding rapamycin to initiate the Cyc8p depletion in cells grown in the presence of mannose, it means that these Flo^-^ cells never flocculated at all. Together, this means our analysis should offer a more accurate insight into the transcriptome of a Cyc8p-depleted cell in the presence and absence of flocculation which would be minimally affected by possible secondary effects.

Although we feel that our experimental strategy has largely controlled for, and negated, the impact of mannose upon our analysis, the possibility remains that mannose could have influenced transcription of some genes. To overcome this caveat, potential future experiments could either repeat our experiment using a non-metabolizable analogue of mannose or use a Cyc8-AA strain in which all the *FLO* genes have been deleted, which would not require mannose addition to inhibit flocculation.

It is interesting to note that following anchor-away of Cyc8p, we see that Tup1p is also lost from at least one well-characterized Tup1-Cyc8 repressed gene, *FLO1* [35]. If the loss of Tup1p seen at *FLO1* following Cyc8-AA occurs at all Cyc8p-regulated genes, it would mean that a Cyc8-AA strain is more similar to a *tup1 cyc8* double mutant than a *cyc8* single mutant. Thus, a Cyc8-AA strain might provide a superior way in which to assess the consequences upon gene transcription due to the loss of the entire Tup1-Cyc8 complex rather than looking in *cyc8* or *tup1* single gene deletion mutants. This may be particularly relevant considering research showing possible independent roles for Tup1p and Cyc8p in regulating gene transcription [4].

A limitation of our study is that it only offers a snapshot of the impact of flocculation upon transcription at a single time point (4 h post-rapamycin treatment). It would be reasonable to predict that repeating our analysis after different times (and extents) of flocculation would yield different results. Indeed, it might be predicted that performing the analysis after a longer time following Cyc8p depletion, in which larger flocs should form, would reveal a distinct cohort of genes subject to an altered influence by flocculation. Ideally, a time-course analysis during flocculation formation would provide the greatest insight into the potentially dynamic influence of flocculation upon gene transcription during the development of the flocculation phenotype.

Even without such a time-course analysis, our data revealed that the flocculation phenotype has a considerable role in governing global gene transcription. Thus, our data suggest that the flocculation survival strategy triggered by environmental and cellular signals, and initially mediated by various transcription factors, is further regulated by the developing phenotype itself.

A better understanding of the potential dynamic interplay between transcription and flocculation might allow global gene transcription to be modulated by altering the flocculation phenotype of cells. This could be valuable to industries using yeast as a production organism whereby strain performance could be enhanced by altering the flocculant properties of the strain to adjust global transcription to give the desired outcome.

## supplementary material

10.1099/mgen.0.001216 Uncited Supplementary Material 1.

10.1099/mgen.0.001216 Uncited Table S1.
