# Supplementary material for: The influence of flocculation upon global gene transcription in a yeast CYC8 mutant
Source: Microb Genom. 2024 Mar 26;10(3):001216. doi: 10.1099/mgen.0.001216 (PMC10995634; doi:10.1099/mgen.0.001216)
Supplement: Uncited Supplementary Material 1. [file mgen-10-01216-s001.pdf]

# The influence of flocculation upon global gene transcription in a yeast *CYC8* mutant

Supplementary Material

Lee *et al*., 2024

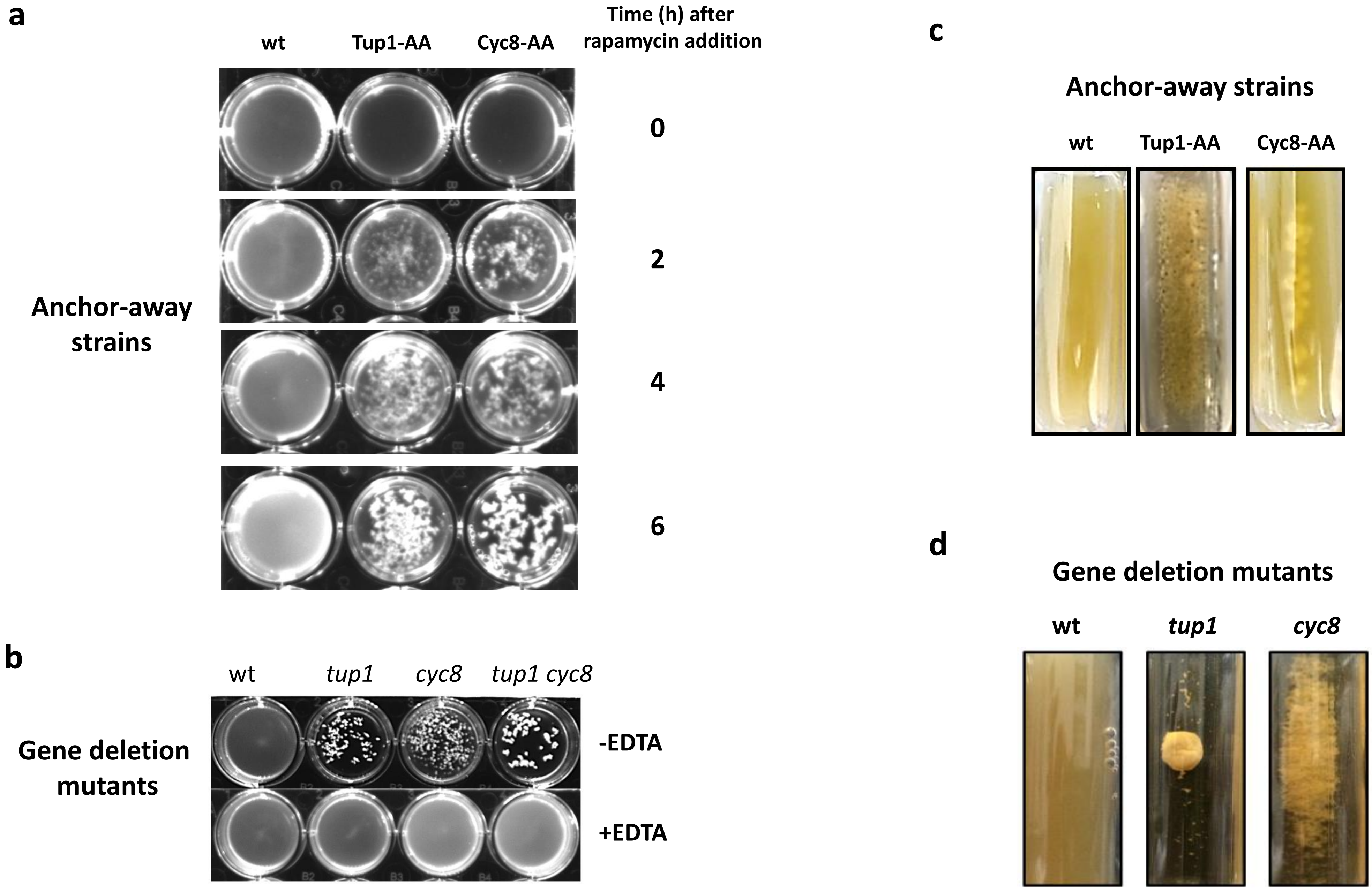


**Supplemental Fig. S1. Flocculation phenotypes in Cyc8 and Tup1 anchor-away strains and in *cyc8* and *tup1* gene deletion mutants.** (a) Images to show the presence or absence of the flocculation phenotype in exponential wt, Tup1-AA, and Cyc8-AA cell cultures following growth for the indicated times (hours) after the addition of rapamycin. (b) Exponentially growing cultures of wt and the gene deletion mutants indicated were photographed in the presence (+EDTA) and absence of EDTA (-EDTA). (c) Images of cultures of the anchor-away strains indicated taken after growth in YPD broth and rapamycin for 24 hours. (d) Images of cultures of wt and the gene deletion mutant strains indicated taken after growth in YPD broth for 24 hours. The images shown in (b) and (d) are from Lee *et al*., 2023 [1]. Together, these data show that in contrast to the *tup1* gene deletion mutant having a stronger flocculation phenotype than a *cyc8* deletion mutant, the Cyc8-AA strain has a stronger flocculation phenotype compared to the Tup1-AA strain, following rapamycin addition.

1. **Lee B, Church M, Hokamp K, Alhussain MM, Bamagoos AA, *et al.*** Systematic analysis of tup1 and cyc8 mutants reveals distinct roles for TUP1 and CYC8 and offers new insight into the regulation of gene transcription by the yeast Tup1-Cyc8 complex. *PLoS Genet* 2023;19:e1010876

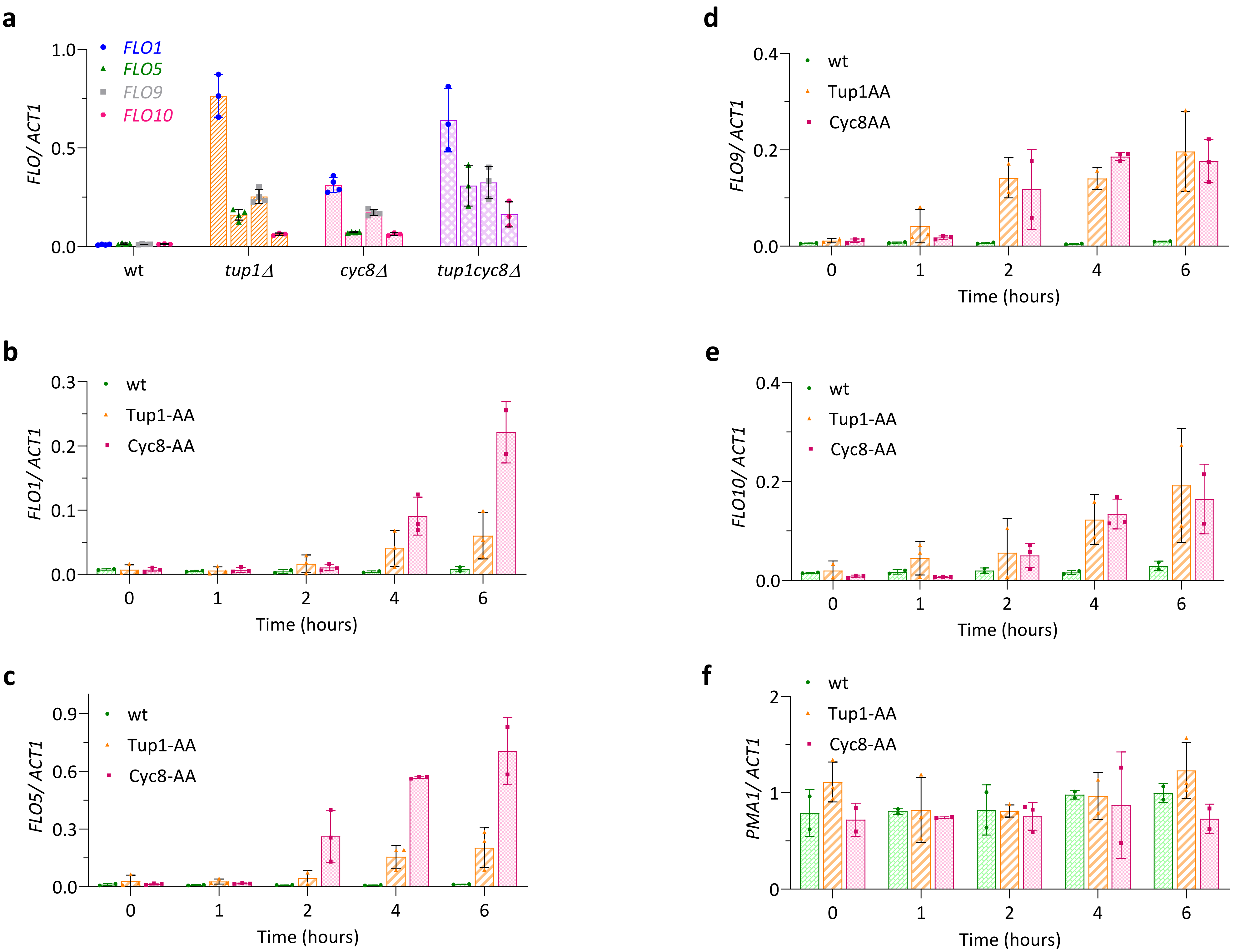


**Supplemental Fig. S2. *FLO* gene transcription in Cyc8 and Tup1 anchor-away strains and in *cyc8* and *tup1* gene deletion mutants.** (A) *FLO1*, *FLO5*, *FLO9* and *FLO10* transcript levels detected by RT-qPCR in wt and the gene deletion mutant strains indicated. RT-qPCR analysis of mRNA levels of (B) *FLO1*, (C) *FLO5*, (D) *FLO9*, (E) *FLO10* and (F) *PMA1* in Cyc8-AA and Tup1-AA cells following the addition of rapamycin. Time (hours) shown after rapamycin addition are indicated. In all graphs, values were normalised to *ACT1* mRNA levels and error bars reflect standard deviation from 3-4 biological replicates. The data shown in (A) is from Lee *et al*., 2023 [1]. *FLO* gene transcription profiles in the anchor away strains following rapamycin addition differ from the levels of *FLO* gene transcription in the gene deletion mutants. Where *FLO1* and *FLO5* were de-repressed to the greatest extent in a *tup1* deletion mutant compared to a *cyc8* deletion mutant, *FLO1* and *FLO5* transcription in the Cyc8-AA strain was significantly higher than that measured for these genes in the Tup1-AA strain. *FLO9* and *FLO10* expression levels are similar in both the Tup1-AA and Cyc8-AA strains over the time course after rapamycin addition. The overall higher levels of *FLO* gene expression in the Cyc8-AA strain compared to that in a Tup1-AA strain are consistent with the stronger flocculation phenotype of the Cyc8-AA strain. Also of note, whereas *FLO1* is the dominantly expressed *FLO* gene in the *cyc8* and *tup1* deletion mutants, *FLO5* is de-repressed to the greatest extent in the Tup1-AA and Cyc8-AA strains following the addition of rapamycin. The *PMA1* gene is a control gene which is not subject to Tup1-Cyc8 regulation; its transcription level remains constant in the Cyc8-AA and Tup1-AA strains at each time point after rapamycin addition.

| Cells | +/- Rapamycin (I ug/ml) | Mannose (mM) | Flocculation |
|---|---|---|---|
| 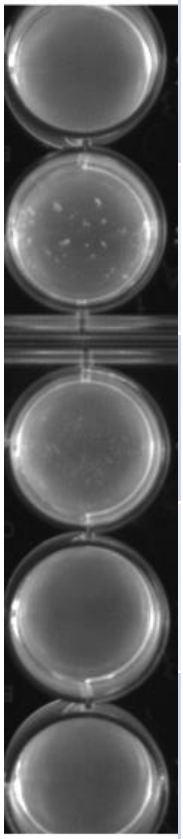 | - | 0 | - |
| | + | 0 | +++ |
| | + | 100 | ++ |
| | + | 200 | + |
| | + | 250 | - |

**Supplemental Fig. S3. Assay to determine the minimal concentration of mannose required to inhibit flocculation.** Cells were grown for four hours (4h) in the presence (+) and absence (-) of rapamycin (1 ug/ml) and mannose at the indicated concentrations (mM). Cell images were taken after 4h, and the '+' sign indicates the relative levels of flocculation visible; '-' indicates the absence of flocculation.

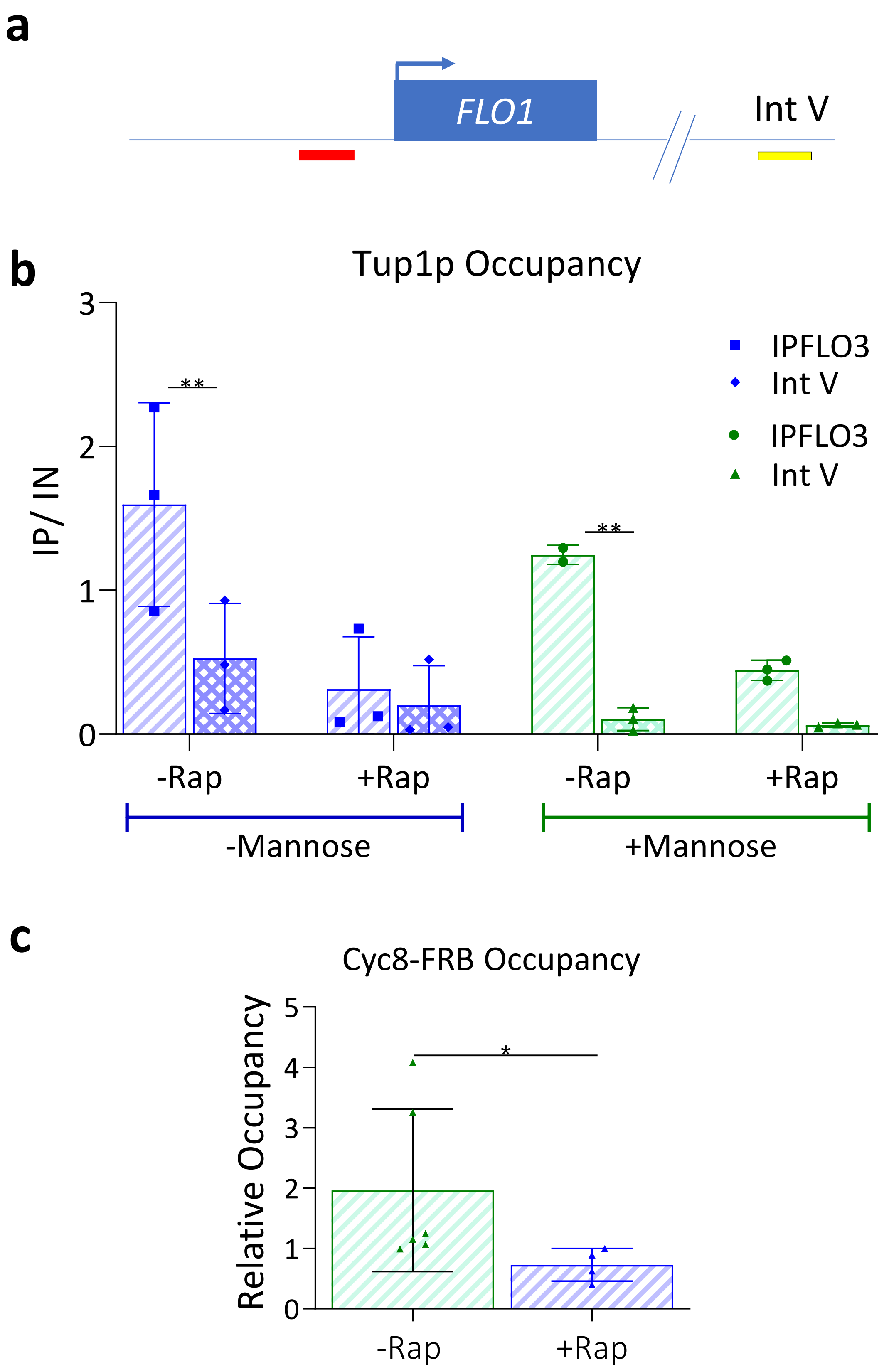


**Supplemental Fig. S4. Tup1p and Cyc8-FRB occupancy at the *FLO1* promoter region after four hours growth in the presence or absence of rapamycin and/or mannose**. (A) Schematic to show the location of the amplicons used for ChIP analysis of Tup1p and Cyc8-FRB. IPFLO3 (red bar) is a positive control region centred around -596 bp upstream of the *FLO1* gene start site (arrow); Int V (yellow bar) is a negative control region located within an intergenic region on chromosome V. (B) Tup1p occupancy (IP/IN) values at the *FLO1* promoter region (IPFLO3) and at the negative control region (Int V) are shown after 4 hours in the presence and absence of rapamycin and/or mannose, as indicated. (C) Cyc8-FRB occupancy (IP/IN) values at IPFLO3 plotted relative to occupancy (IP/IN) at Int-V after 4 hours in the presence and absence of rapamycin, and in the absence of mannose. Mean and standard deviation are shown from 4-6 biological replicates; asterisk represents a p-value of $**=p \leq 0.005$ obtained from a One-Way ANOVA analysis. As can be seen in C, although there was a significant depletion of Cyc8-FRB from the *FLO1* promoter 4h after rapamycin addition, we consider the ChIP of Cyc8-FRB to be of low sensitivity. Thus, we opted to perform Tup1p ChIP as the most robust way of monitoring the anchor away of Cyc8p in the presence and absence of mannose (B), consistent with previously published data that showed that Tup1p is also rapidly lost (30 min post-rapamycin treatment) from the *FLO1* promoter following Cyc8p anchor away.

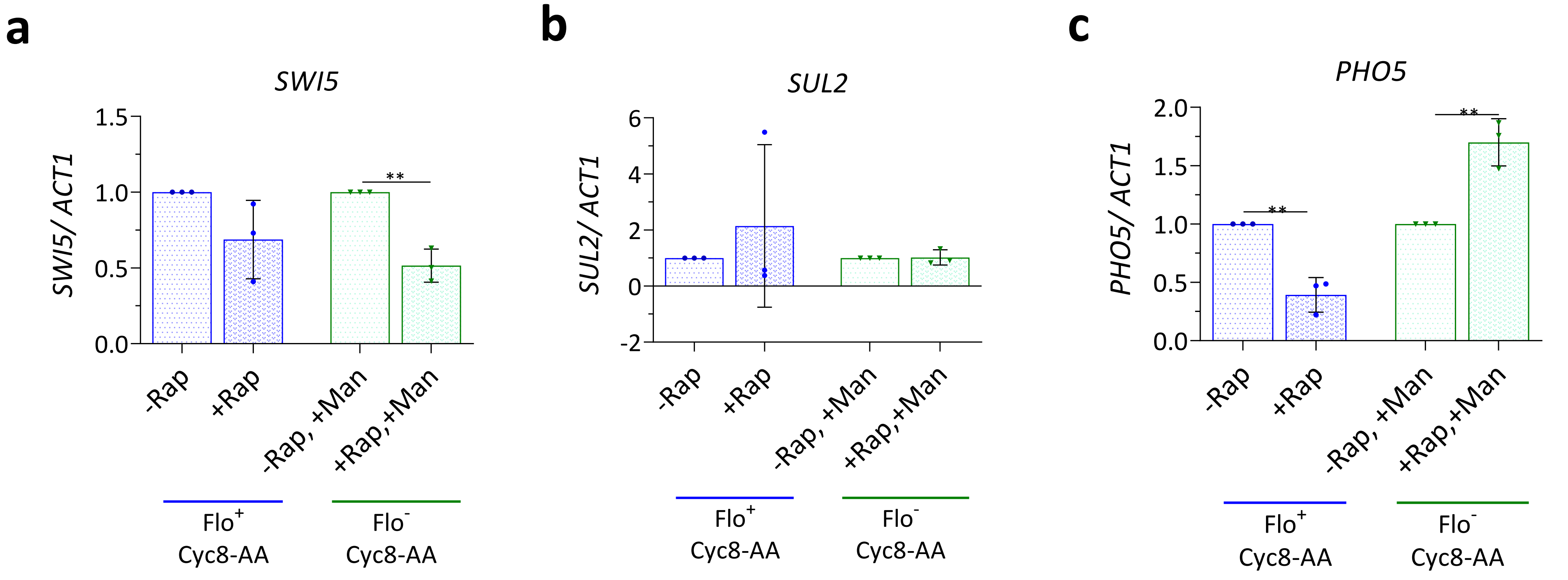


**Supplemental Fig. S5. Validation of the RNA-seq data shown in the JBrowse images for *SWI5*, *SUL2* and *PHO5 mRNA* levels.** *SWI5, SUL2* and *PHO5* mRNA transcript levels measured relative to *ACT1* mRNA levels using RT-qPCR in Flo$^{+}$ and Flo$^{-}$ Cyc8-AA experiments. For both Flo$^{+}$ and Flo$^{-}$ anchor away experiments, the mRNA values of each target gene after rapamycin addition (+Rap) were further normalised to the mRNA levels in the absence of rapamycin (-Rap), which were set at a value of 1. Error bars reflect standard deviation from 3 biological replicates (**) represents a p-value of $p<0.005$ determined by a One-way ANOVA analysis).

**Supplementary Table S1. Oligonucleotides used in study.**

| Name: | Sequence (5’-3’): | Description: | Distance relative to ATG: |
|---|---|---|---|
| *ACT1* ORF-F | GAGGTTGCTGCTTTGGTTATTGA | *ACT1* transcription | +318 |
| *ACT1* ORF-R | ACCGGCTTTACACATACCAGAAC | | |
| *FLO1* RT-F | TACCACCACAGACGGGTTCT | *FLO1* transcription | +481 |
| *FLO1* RT-R | CAACAGTTGAACGCGGTTGC | | |
| *FLO5* RT5'-F2 | GGATGGAAGTCTCCCTGACA | *FLO5* transcription | +635 |
| *FLO5* RT5'-R2 | GGAAACGGCATTGGAGTAAA | | |
| *FLO9* RT5'-F | TCGTCACATTGCTGGGATTA | *FLO9* transcription | +105 |
| *FLO9* RT5'-R | TGCTGCATTCGAATATGTGG | | |
| *FLO10* RT-F | GCGGTTAGTTCTGACATCGAAAAT | *FLO10* transcription | +3164 |
| *FLO10* RT-R | TTTTGTCTCAGCAGCCTCTGAA | | |
| *SUT1*_RT_F | CATGGTCAATGAAGACGCTAATG | *SUT1* transcription | +395 |
| *SUT1*_RT_R | ACTAGTAGATGTGGCAGAGGA | | |
| *SWI5*_RT_F | GACTCCCTCATTTCACCAAAGA | *SWI5* transcription | +930 |
| *SWI5*_RT_R | TTGGAGTGAAGGGCGTAATAAA | | |
| *PHO5*_RT-F | CGACAAGATTGGTACCCAAAAA | *PHO5* transcription | +136 |
| *PHO5*_RT-R | CTTCAGGCAAATCACGAGAAAT | | |
| *SUC2* RT486-F | AGCTGCCAACTCCACTCAAT | *SUC2* transcription | +486 |
| *SUC2* RT486-R | ATTTGGCAGCCGTCATAATC | | |
| *DIP5*_RT-F | CTCGTTCATCCACCTCTACATCAC | *DIP5* transcription | +204 |
| *DIP5*_RT-R | AACCGCGTGTTTTCGTCTTT | | |
| *SUL2*_ORF_F | AAGGGAGAACGACCCTGAATC | *SUL2* transcription | +2288 |
| *SUL2*_ORF_R | TGGCCTGTCATCTCTTTCAACA | | |
| *PMA1*_ORF_F | GAAAAAGAATCTTTAGTCGTTAAGTTCGTT | *PMA1* transcription | +322 |
| *PMA1*_ORF_R | AATTGGACCGACGAAAAACATAA | | |
| *INT-V* | TAAGAGGTGATGGTGATAGGCGT | Control region (ChIP) | N/A |
| *INT-V* | CCCTCGGGTCAAACACTACAC | | |
| IPFLO3-F | GCTTCCAGTATGCTTTCACG | *FLO1* Promoter (ChIP) | -585 |
| IPFLO3-R | GCCTACGTATTCTCCGTCAC | | |
